# Cytotoxic CD8^+^ Temra cells show loss of chromatin accessibility at genes associated with T cell activation

**DOI:** 10.3389/fimmu.2024.1285798

**Published:** 2024-02-02

**Authors:** Lehte Türk, Igor Filippov, Christian Arnold, Judith Zaugg, Liina Tserel, Kai Kisand, Pärt Peterson

**Affiliations:** ^1^ Institute of Biomedicine and Translational Medicine, University of Tartu, Tartu, Estonia; ^2^ Qiagen Aarhus A/S, Aarhus, Denmark; ^3^ European Molecular Biology Laboratory, Structural and Computational Biology Unit, Heidelberg, Germany

**Keywords:** aging, T cells, terminal differentiation, chromatin changes, single-cell, transcriptomics

## Abstract

As humans age, their memory T cell compartment expands due to the lifelong exposure to antigens. This expansion is characterized by terminally differentiated CD8^+^ T cells (Temra), which possess NK cell-like phenotype and are associated with chronic inflammatory conditions. Temra cells are predominantly driven by the sporadic reactivation of cytomegalovirus (CMV), yet their epigenomic patterns and cellular heterogeneity remain understudied. To address this gap, we correlated their gene expression profiles with chromatin openness and conducted single-cell transcriptome analysis, comparing them to other CD8^+^ subsets and CMV-responses. We confirmed that Temra cells exhibit high expression of genes associated with cytotoxicity and lower expression of costimulatory and chemokine genes. The data revealed that CMV-responsive CD8^+^ T cells (Tcmv) were predominantly derived from a mixed population of Temra and memory cells (Tcm/em) and shared their transcriptomic profiles. Using ATAC-seq analysis, we identified 1449 differentially accessible chromatin regions between CD8^+^ Temra and Tcm/em cells, of which only 127 sites gained chromatin accessibility in Temra cells. We further identified 51 gene loci, including costimulatory *CD27*, *CD28*, and *ICOS* genes, whose chromatin accessibility correlated with their gene expression. The differential chromatin regions Tcm/em cells were enriched in motifs that bind multiple transcriptional activators, such as Jun/Fos, NFkappaB, and STAT, whereas the open regions in Temra cells mainly contained binding sites of T-box transcription factors. Our single-cell analysis of CD8^+^CCR7^lo^CD45RA^hi^ sorted Temra population showed several subsets of Temra and NKT-like cells and CMC1^+^ Temra populations in older individuals that were shifted towards decreased cytotoxicity. Among CD8^+^CCR7^lo^CD45RA^hi^ sorted cells, we found a decreased proportion of IL7R^+^ Tcm/em-like and MAIT cells in individuals with high levels of CMV antibodies (CMV^hi^). These results shed new light on the molecular and cellular heterogeneity of CD8^+^ Temra cells and their relationship to aging and CMV infection.

## Introduction

In aged individuals, the human T cell compartment becomes enriched in CD8^+^ terminally differentiated effector memory T cells, named Temra, in which accumulation has been associated with increased immunosenescence impacting adaptive immune responses (1–3). Temra cells have low expression of chemokine receptor CCR7 but re-express CD45RA marker that is characteristic to naïve T cells (Tna) (4). Typically, the expression of CD45RA marker is used to distinguish Temra from central and effector memory T cells (Tcm/em) that have CD45RO, another splice variant of the *PTPRC* gene (5). CD8^+^ Temra cells gradually lose costimulatory CD27 and CD28 markers, gain NK cell-like phenotype and show high production of cytotoxic and inflammatory molecules (6–8). However, these phenotypic and functional changes in the late differentiation of T cells may represent a biological adaptation of the immune system to changing host environments over years, such as an increase in chronic infections (9).

The well-known causes of terminal T cell differentiation are recurrent viral and bacterial infections and the presentation of misfolded proteins of apoptotic cells engulfed by antigen-presenting cells (10). Chronic infection of human cytomegalovirus (CMV) is considered the primary driving factor in CD8^+^ Temra cell expansion and repertoire inflation (11). The infection goes unnoticed in healthy individuals, however, based on antibody responses, the infection rate can reach over 90% of the population (12, 13). Recently a strong activation of the *IFNG* gene and combined expression of *TNFRSF9*, *XCL1*, *XCL2*, and *CRTAM* were found as the characteristic genes of CMV-responsive CD8^+^ T cells (14).

A distinct feature of aging is an increased heterogeneity between individuals and elevated cell-to-cell variability in chromatin modifications (15). Age-associated epigenetic changes are more pronounced in CD8^+^ compared to CD4^+^ T cells, as CD8^+^ T cells from old adults, irrespective of their differentiation state, display greater reduced accessibility to genes of basic biological function (16). With age, CD8^+^ T cells exhibit a shift from naïve cells toward memory-associated chromatin patterns and loss of chromatin accessibility at promoters (17). Memory CD8^+^ T cells undergo chromatin remodeling with aging as they show systematic chromatin closing at promoters and enhancers such as the silencing of the *IL7R* gene and the IL-7 signaling pathway genes and aging-related loss in the binding of NF-κB and STAT factors (18).

While the epigenetic differences between Tna and Tem cells are well documented (15–18), the epigenetic differences between CD8^+^ memory and late-differentiated Temra cells are less studied. Rodriguez et al. showed that CD8^+^ Tem and Temra cells have remarkable similarities at the transcriptional and epigenetic levels and proposed that Temra cells may not be a truly distinct population and only represent the aged effector memory T cell population (19). A recent study on memory T cells identified epigenomic and transcriptomic changes in genes of cytotoxicity, metabolism, and self-renewal, and associated these with specific transcriptional factor motifs (20). However, other studies have shown not only a difference between Tem and Temra cells but also identified substantial heterogeneity within Temra population. Based on the surface marker CD57 Verma et al. separated Temra cells into terminal CD57^+^ and less differentiated CD57^-^ subset with high proliferative capacity and differentiation plasticity (21). In addition to CD57, co-signaling markers CD27 and CD28 have been used to separate Temra populations (22), and CD27^-^CD28^+^CD8^+^ Temra subpopulation was related to mortality among octogenarians (23). A distinct CD16^+^ Temra CD8^+^ T cell subpopulation was increased in smokers and kidney transplantation patients and was characterized by high levels of proinflammatory cytokine secretion and cytotoxic activity (24, 25). Furthermore, granzyme markers GZMB and GZMK have been used to distinguish Tem and Temra subpopulations (26).

We here investigated the transcriptome and chromatin accessibility of CD8^+^ Temra cells in comparison to other CD8^+^ subsets and their relation to CMV responses. We analyzed the transcriptional regulator occupancy in open chromatin regions of Temra cells and studied its sorted cell population heterogeneity by single-cell transcriptome analysis (**Supplementary Figure S1**).

## Results

### Temra cells upregulate genes with NK cell and cytotoxicity function

**Figure 1:**
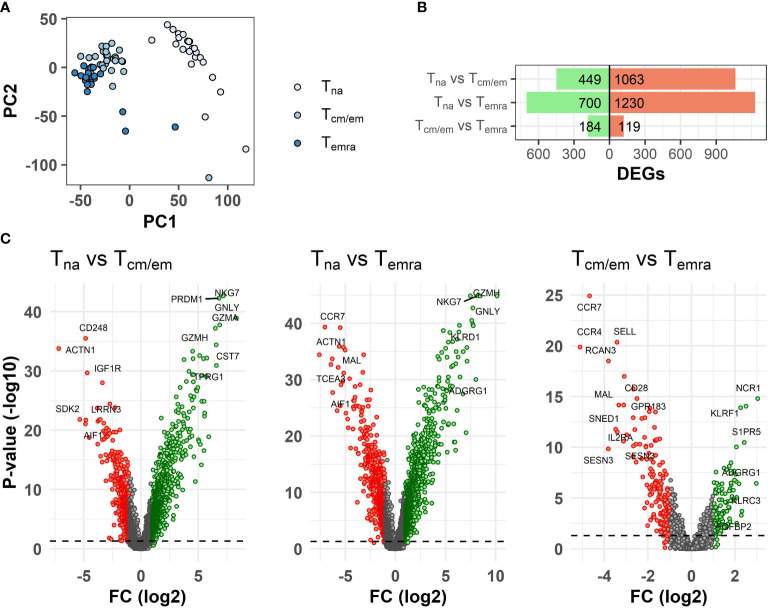
Differences in transcriptome between CD8^+^ T cell subsets. **(A)** Principal component analysis (PCA) of gene expression levels of T cell subsets. **(B)** Summary of differentially expressed genes between T cell subsets. **(C)** Volcano plots of differently expressed genes (DEGs) between T cell subsets. The volcano plots show log2 fold change (x-axis) versus -log10 FDR-adjusted p-value (y-axis). Genes that are not differentially expressed (fold change < abs (2) and/or FDR-adjusted p-value≥0.05) are colored grey. The horizontal dashed line marks FDR-adjusted p-value=0.05. Labels were added to the top most differentially expressed genes. Tna, naïve T cells; Tcm/em, central and effector memory T cells; Temra, terminally differentiated effector memory T cells.

**Figure 2:**
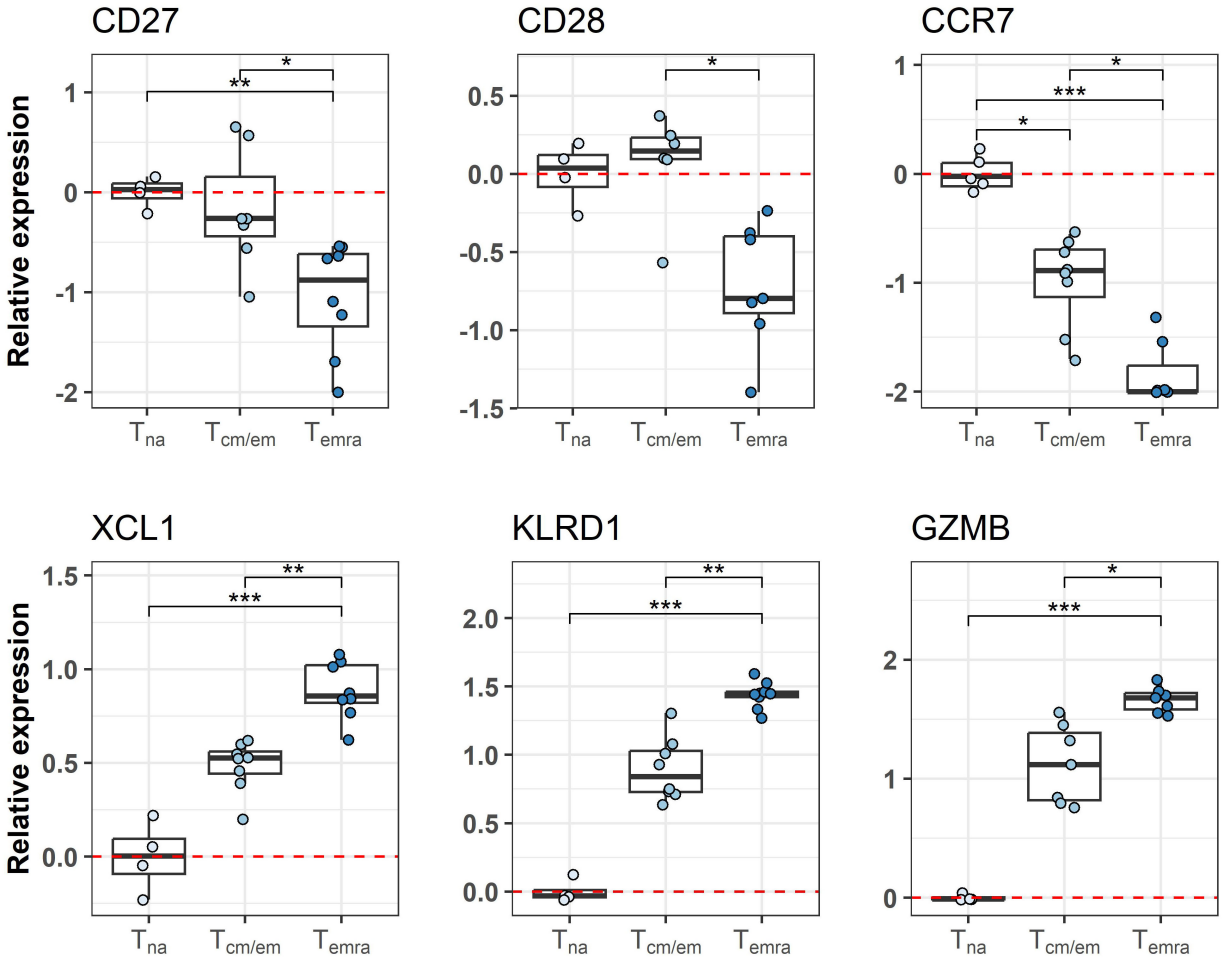
Differences in transcriptome between CD8^+^ T cell subsets is validated by qPCR. Relative expression of genes is represented as boxplots. Tna cells are chosen as the reference group as indicated by red dashed line. * Indicates p-value ≤0.05, ** indicates p-value ≤0.01, *** indicates p-value ≤0.001. Tna, naïve T cells; Tcm/em, central and effector memory T cells; Temra, terminally differentiated effector memory T cells.

We used CCR7 and CD45RA markers to separate CCR7^hi^ CD45RA^hi^ naïve (Tna), CCR7^hi^/CCR7^lo^ CD45RA^lo^ central and effector memory (Tcm/Tem) and CCR7^lo^ CD45RA^hi^ terminal effector memory CD45RA^+^ (Temra) cells from ≥65-year-old individuals (**Table 1**) and studied their genome-wide gene expression by microarray. The PCA analysis of all gene signals separated the CD8^+^ T cell subsets, from which the Tna cell population was notably different from Tcm/em and Temra populations. (**Figure 1A**). CD8^+^ Tna cells were distinct from CD8^+^ Tcm/em and Temra cells in many differentially expressed genes. Altogether, 449 and 700 genes were upregulated, whereas 1063 and 1230 genes were downregulated in Tna compared to Tcm/em and Temra cells, respectively (**Figure 1B**, **Supplementary Table S1**). Tcm/em and Temra subsets were closer in their expression profiles as they differentially expressed only 303 genes (184 of those upregulated in Tcm/em and 119 upregulated in Temra). Both Tcm/em and Temra cells showed upregulation of multiple membrane receptors associated with NK cell activity (*KLRG1*, *KLRF1*, *KLRB1*, *KLRC1*, *KLRC2*, *KLRD1*, *NKTR*, *NKG7*, *FCR3A*, KIR2D family, *CD244*), and molecules involved in cytotoxicity (*PRF1*, *GZMA*, *GZMB*, *GZMH*, *GZMK*, *GNLY*) (**Figure 1C**, **Supplementary Table S1**). Compared to Tcm/em, Temra cells had higher expression of the genes associated with NK cell and cytotoxicity functions (*KLRG1*, *KLRF1*, *KLRC1*, *CD244*, *PRF1*, *GZMB*, *GZMH*, *GNLY*). Furthermore, the *NCR1* gene, known as NK-cell marker NKp46, was the top upregulated gene in Temra population. The chemokine receptors *CCR4* and *CCR7*, and *SESN3*, a stress-induced regulator of the AMPK-mTORC1 pathway were downregulated in Temra cells (**Figure 1C**). The gene ontology analysis also showed that Temra cells upregulate genes that are enriched in cytotoxicity-related pathways (**Supplementary Figure S2**). We confirmed the downregulation of costimulatory molecules *CD27* and *CD28*, and chemokine receptor *CCR7* as well as upregulation of *XCL1*, *KLRD1*, and *GZMB* in Temra cells by real-time PCR (**Figure 2**). The results show the increased NK cell-like and cytotoxicity function in Temra population.

**Table 1:** Gender, age and CMV status of the study participants.

Age group	Total	Male	Female	CMV^hi^	CMV^lo^
Transcriptome and epigenome analysis					
**65-70**	6	2	4	6	0
**71-80**	16	4	12	16	0
**81-90**	10	0	10	10	0
**Total**	31	6	26	31	0
Single-cell analysis					
**21-34 (young)**	4	0	4	1	3
**>65 (old)**	7	2	5	3	4
**Total**	11	2	9	4	7

### The transcriptome profile of CMV-responsive T cells (Tcmv) is closer to Tcm/em and Temra cells but different from Tna

**Figure 3:**
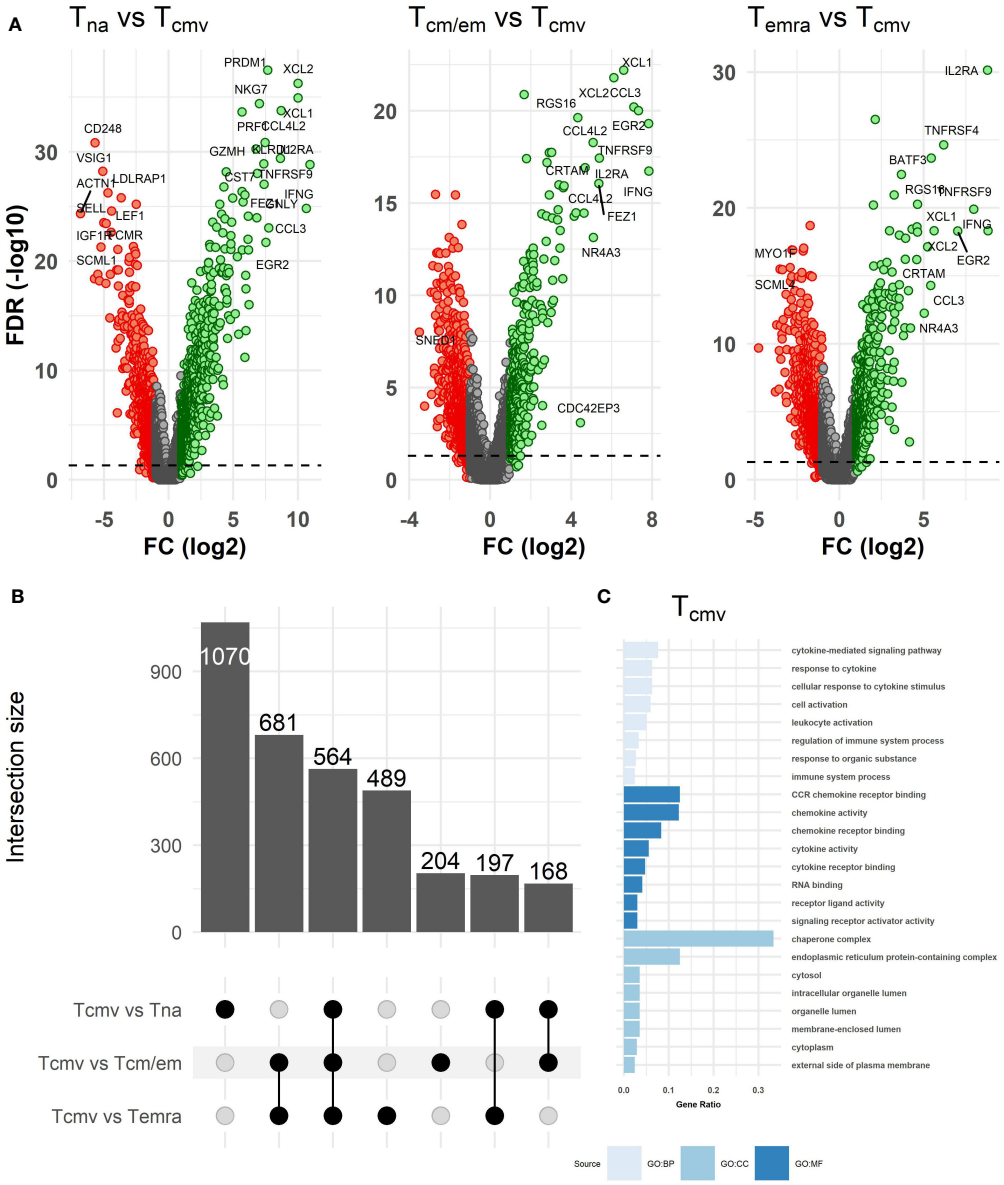
Differences in transcriptome between Tcmv and resting (Tna, Tcm/em and Temra) CD8^+^ T cell subsets. **(A)** Volcano plots of differentially expressed genes between Tcmv and resting T cell subsets. The volcano plots show log2 fold change (x-axis) versus -log10 FDR-adjusted p-value (y-axis). Genes that are not differentially expressed (fold change < abs (2) and/or FDR-adjusted p-value≥0.05) are colored grey. The horizontal dashed line marks FDR-adjusted p-value=0.05. Labels were added to the top most differentially expressed genes. **(B)** Upset plot showing the overlap of differentially expressed genes (DEGs) in CD8^+^ T cell subsets between all pairwise comparisons. The bar plots on the left show the total number of DEGs in each comparison, while bar plots on the top show DEGs that are shared between pairwise comparisons or are differentially expressed only in one comparison (uniquely differentially expressed). **(C)** GO enrichment analysis of genes upregulated in Tcmv cells. The bar size reflects the gene count/term size (gene ratio). BP, biological process; CC, cell component; MF, molecular function. Tna, naïve T cells; Tcm/em, central and effector memory T cells; Temra, terminally differentiated effector memory T cells; Tcmv, CMV-responsive CD8^+^ T cells.

As chronic CMV infection is considered the main driver of Temra cell accumulation and TCR repertoire inflation in old individuals, we studied the proportion of CMV pp65-responsive T cells (Tcmv) from CD8^+^ T cells after their stimulation with an overlapping peptide pool spanning the sequence of the pp65 antigen. We identified Tcmv cells by measuring activation induced markers CD154 and CD137 and correlated their numbers to the Temra counts. The ratio of Tcmv cells ranged from 0.26% to 26.3% (median:1.46%, IQR: 2.88%) of the total CD8^+^ T cell fraction (**Supplementary Figure S3**); however, we found no correlation between Temra or Tcm/em cell proportions and pp65-responsive cell numbers (**Supplementary Figure S4A**). The T cells specific for the viral protein pp65 (peptide pool) were a mixed population of Tcm/em and Temra cells (75% - mixed, 20% mostly Temra, 5% - mostly Tcm/em; **Supplementary Figures S4B, C**). The transcriptome profile of Tcmv cells was closer to Tcm/em and Temra cells and distinct from the Tna population as estimated by differential gene expression number and fold changes (**Figures 3A, B**, **Supplementary Table S2**). There were altogether 564 genes that were differentially expressed in resting T cell subsets compared to Tcmv cells (**Figure 3B**). The transcriptome of CD8^+^ Tcmv cells showed strong upregulation of *IFNG* and *TNF* genes, activation-specific T cell markers, *TNFRSF9*, *CD25*, and *TNFRSF4*, chemokines (*XCL1*, *XCL2*, *CCL3*, *CCL4L2*, *CCL20*, *CCL1*), *CRTAM* and transcription factors *EGR2* and *NR4A3* (**Figure 3A**, **Supplementary Figure S5**, **Supplementary Table S2**). Expectedly, anti-apoptotic genes *BCL2L1* and *BCL2A1* were upregulated in Tcmv cells. The downregulated genes were functionally diverse and did not form a common group, underlining the activated status of Tcmv cells. However, most downregulated genes in Tcmv cells were *SEPT9*, *PLAC8* and *FCMR* (**Supplementary Figure S5**). Tcmv cells had also lower expression of *SESN3* when compared to Tna and Tcm/em populations. The gene ontology analysis showed that CD8^+^ Tcmv cells upregulated the genes associated with cytokine and chemokine pathways (**Figure 3C**). We also compared our CMV-induced gene data to recently published dataset of stimulated and non-stimulated Tcm, Tem and Temra cells (20). After subtracting the activation-induced genes from the list of differentially expressed genes (Tcmv vs Temra, and Tcmv vs Tem/cm), we observed that the top genes that differentiate Tcmv from the two memory cell subtypes overlapped to a great extent (**Supplementary Figure S6**). Thus, our results confirm that Tcmv cells are transcriptionally more similar to Tcm/em and Temra populations than to Tna.

### Temra cells are epigenetically more closed compared to Tcm/em cells

**Figure 4:**
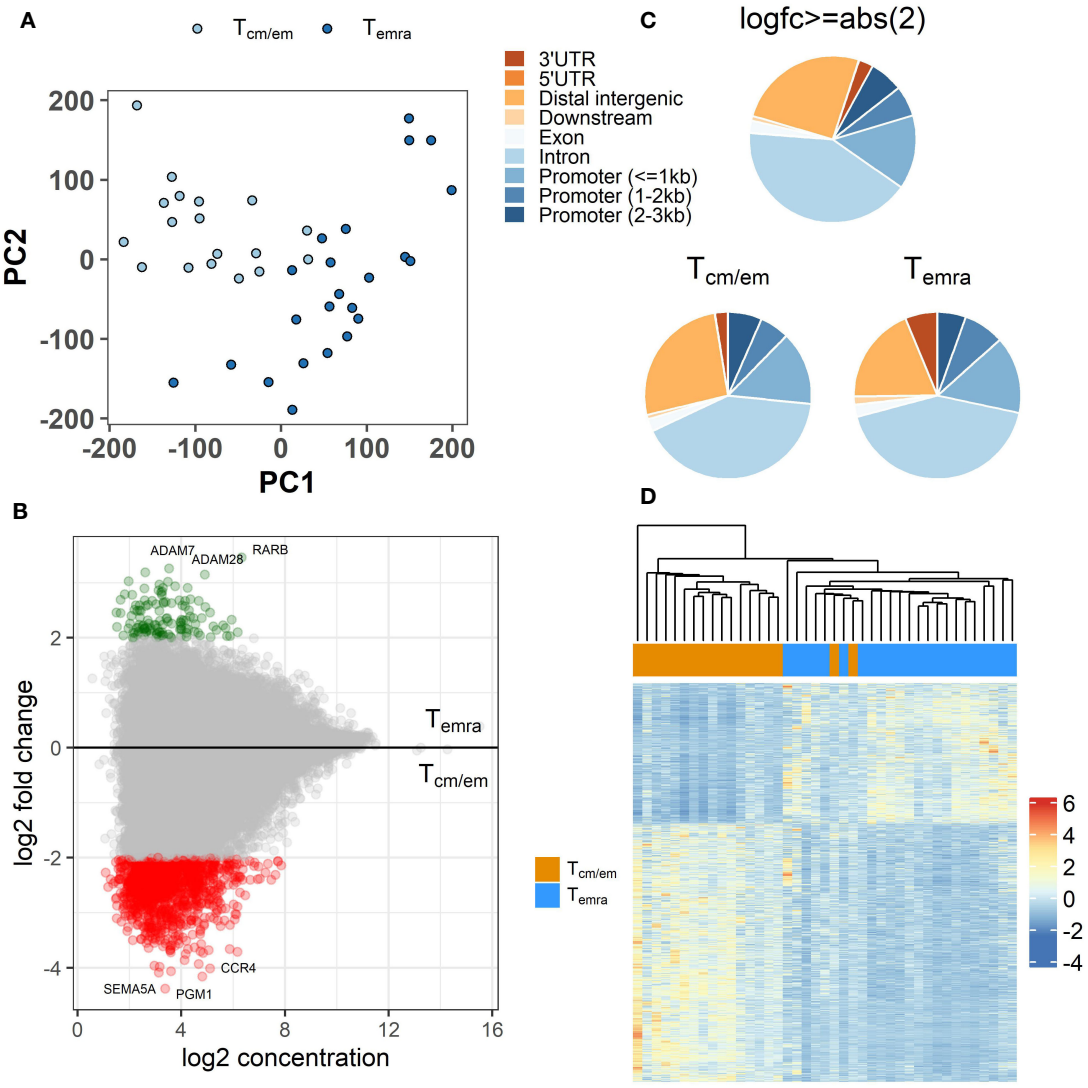
Differences in epigenome between CD8^+^ Temra and Tcm/em cells. **(A)** Principal component analysis (PCA) of ATAC-seq peaks. **(B)** Differentially accessible sites between Temra and Tcm/em cells plotted as log2 fold change versus the mean of normalized counts. Log2 fold change ≥2 (more open in Temra) and ≤-2 (more open in Tcm/em) are colored green and red, respectively. **(C)** Location of differentially accessible genes: 1) all regions (log2 fold change ≥abs (2)), 2) regions more open in Tcm/em cells (log2 fold change ≥2) 3) regions more open in Temra cells (log2 fold change ≤-2). **(D)** Heatmap showing z-scores for differentially accessible ATAC-seq peaks with adjusted FDR-adjusted p-value <0.05 (10609) across Tem/cm and Temra samples. Tcm/em, central and effector memory T cells; Temra, terminally differentiated effector memory T cells.

During the late stages of their differentiation, CD8^+^ T cells undergo epigenetic changes in their chromatin structure (27). We used ATAC-seq to explore the chromatin changes occurring in terminal differentiation from Tcm/em to Temra cells. We identified 63920 peaks that were present in at least 2 individuals. The PCA plot of the ATAC-seq peaks showed more uniform clustering of Tcm/em and higher heterogeneity of Temra samples (**Figure 4A**). We identified 1322 peaks in 985 individual genes, which showed decreased chromatin accessibility in Temra compared to Tcm/em population (**Figure 4B**, **Supplementary Table S3**). In contrast, only 127 chromosomal regions, annotated to 94 genes, gained openness in Temra cells, indicating decreasing chromatin accessibility during the CD8^+^ T cell terminal differentiation. Moreover, there were 187 gene regions in Tcm/em and 20 in Temra cells that included more than one open region per gene locus indicating lower chromatin accessibility in Temra cells on single gene levels. In total, 14.4% of the differentially accessible regions were located within 1 kb, and another 6% were approximately 1-2 kb from promoters. Most differentially accessible peaks were distal intergenic (25.5%) or located in introns (41.5%) (**Figure 4C**). The clustering of all 10609 differential peaks with FDR <0.05 separated Tcm/em and Temra populations into two distinct groups (**Figure 4D**).

The top open chromatin regions in Temra cells were the genomic loci of metalloproteases *ADAM7* and *ADAM28* in chromosome 8 and nuclear transcriptional regulator *RARB* in chromosome 3 (**Supplementary Table S3**). ADAM28 has been implicated in the shedding of surface target proteins, such as FASL and CD40L in lymphocytes. In Tcm/em cells the top accessible genes were *SEMA5A*, *PGM1* and *CCR4* (**Supplementary Table S3**). However, we found none of these top genes, except *CCR4*, to be differentially expressed in the CD8^+^ T cell subsets. The open chromatin regions in Tcm/em cells were associated with several pathways like the movement of cells, intracellular signal transduction, and vesicle-related pathways.

Taken together, in our genome-wide analysis we found the Temra cells to be epigenetically more closed than Tcm/em cells as memory cells had approximately 10-fold more open chromatin regions than Temra cells.

### Transcriptome-epigenome correlation reveals several immunologically relevant genes

**Figure 5:**
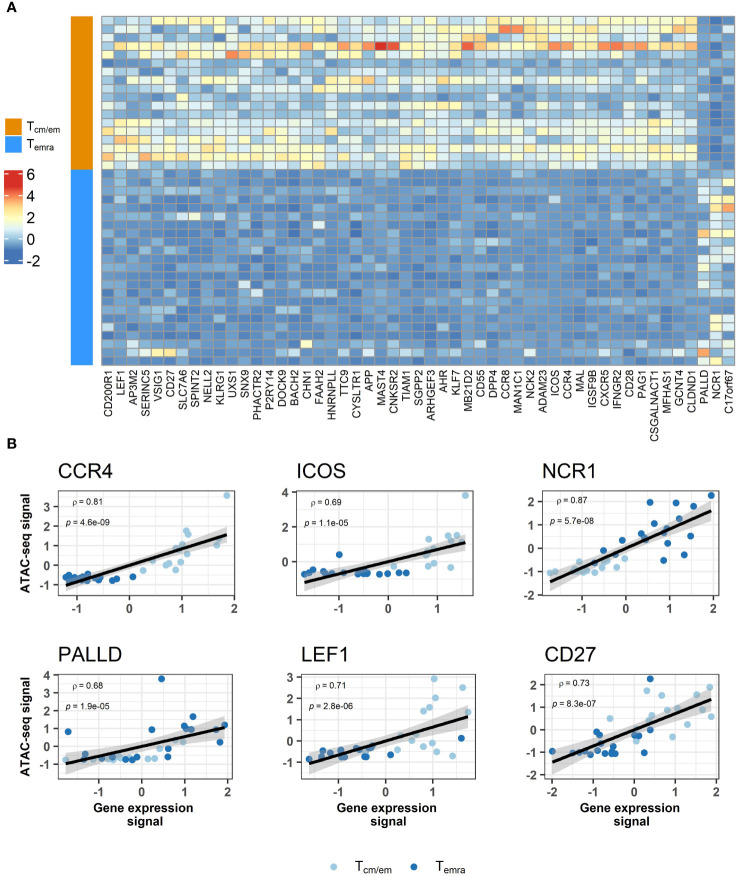
Comparison of transcriptome and epigenome results between CD8^+^ Temra and Tcm/em cells. **(A)** Heatmap showing z scores for differentially accessible ATAC-seq peaks annotated to genes that overlap with transcriptome analysis. For genes that were associated with more than one differentially accessible region, peak with highest difference between two groups is shown. Chromatin accessibility and gene expression correlation among subjects shown as scatterplots **(B)**. Values are centered and scaled (z-scores). Tcm/em, central and effector memory T cells; Temra, terminally differentiated effector memory T cells.

We next tested whether changes in chromatin accessibility directly correlated with gene expression and found 51 genes with overlap in their transcriptome and epigenome changes. The majority of the genes were downregulated in Temra cells, in agreement with decreased chromatin accessibility of regions associated with these genes (**Table 2**, **Figures 5A, B**, **Supplementary Figure S7**). These included well-known markers of memory T cell subset such as costimulatory molecules *CD28*, *CD27*, *ICOS* and chemokine receptors *CXCR5*, *CCR8*, *CCR4* (**Figure 5B**, **Supplementary Figure S7**, **Table 2**). Tcm/em population showed higher expression and chromatin accessibility in several genes encoding transcription factors, such as *LEF1*, *KLF7*, *BACH2*, and *AHR* (**Table 2**). In Temra cells, we found increased accessibility in the promoter region of *NCR1* and *PALLD*, the two genes, for which expression was also upregulated in comparison to Tcm/em cells (**Figure 5B**, **Supplementary Figure S7**, **Table 2**). The upregulation of *NCR1* in Temra cells is in line with the development of NK-like phenotype in Temra cells. *PALDD* is a widely expressed protein needed for the formation of the actin cytoskeleton and supports cell motility. Although the role of the *PALLD* gene in Temra cells remains unknown, it acts as a biomarker in heart-infiltrating immune cells of patients with ischemic cardiomyopathy (28).

**Table 2:** Gene list of overlapping results between gene expression and ATAC-seq analysis.

Gene name	Fold change (gene expression)	Log2 fold change (ATAC-seq)	Direction of change
PALLD	2.12	2	Tcm/em ↓
CD55	3.73	2.1	Tcm/em ↑
DPP4	3.39	2.12	Tcm/em ↑
AP3M2	2.54	2.11	Tcm/em ↑
PAG1	3.27	2.16*	Tcm/em ↑
ADAM23	2.31	3.23*	Tcm/em ↑
MAL	10.02	3.08*	Tcm/em ↑
TIAM1	2.75	3.28*	Tcm/em ↑
AHR	2.92	2.94*	Tcm/em ↑
LEF1	4.85	2.25	Tcm/em ↑
VSIG1	5.64	2.32*	Tcm/em ↑
CLDND1	2.73	2.86	Tcm/em ↑
ARHGEF3	2.14	2.72*	Tcm/em ↓ ↑
CD27	4.44	2.12	Tcm/em ↑
MAN1C1	3.11	2.27	Tcm/em ↑
IFNGR2	2.84	2.61	Tcm/em ↑
NCK2	2.37	2.21*	Tcm/em ↑
KLRG1	3.09	2.16	Tcm/em ↓↑
MAST4	3.83	2.31*	Tcm/em ↑
NELL2	6.35	2.67*	Tcm/em ↑
P2RY14	3.72	2.49	Tcm/em ↑
CCR4	34.48	4.01*	Tcm/em ↑
CXCR5	2.72	2.64	Tcm/em ↑
SGPP2	4.37	2.75*	Tcm/em ↑
DOCK9	2.74	3.03	Tcm/em ↑
CCR8	2.1	2.84*	Tcm/em ↑
SERINC5	2.74	2.27*	Tcm/em ↑
FAAH2	6.37	2.49*	Tcm/em ↑
KLF7	2.18	2.29	Tcm/em ↑
CSGALNACT1	2.18	2.56*	Tcm/em ↑
CD28	8.45	2.39*	Tcm/em ↑
PHACTR2	2.23	2.25*	Tcm/em ↑
NCR1	8.25	2.67*	Tcm/em ↓
UXS1	2.15	2.71	Tcm/em ↑
GCNT4	3.16	3.16*	Tcm/em ↑
MB21D2	2.34	3.24*	Tcm/em ↑
IGSF9B	7.77	2.22*	Tcm/em ↑
HNRNPLL	2.49	2.56	Tcm/em ↑
ICOS	3.03	3.22	Tcm/em ↑
CD200R1	2.41	2.19	Tcm/em ↑
CHN1	2.38	2.65	Tcm/em ↑
MFHAS1	2.45	2.41	Tcm/em ↑
TTC9	4.69	2.59*	Tcm/em ↑
BACH2	2.74	2.2*	Tcm/em ↑
SPINT2	2.45	2.18*	Tcm/em ↑
APP	2.5	2.51	Tcm/em ↑
C17orf67	2.03	2.06	Tcm/em ↑↓
SLC7A6	2.34	2.02	Tcm/em ↑
CNKSR2	2.41	2.13*	Tcm/em ↑
CYSLTR1	4.88	2.11	Tcm/em ↑
SNX9	3.68	2.01	Tcm/em ↑

*More than one differentially accessible region associated with respective gene. Highest log2 fold change is shown.

↑ - Gene is up-regulated/has higher chromatin accessibility in Tcm/em compared to Temra.

↓ - Gene is down-regulated/has lower chromatin accessibility in Tcm/em compared to Temra.

### Differences in chromatin openness between CMV high and low responders

In addition, we compared the donors based on their T cell response to CMV antigens. We divided the individuals into two groups of higher or lower responders (36% and 64% of individuals, respectively) based on their mean response to CMV antigens (2.14%) and searched for chromatin regions that are differentially open in Temra population between those groups. We identified 35 chromatin regions [15 of them had log2 fold change ≥abs (2)], many of them in gene promoters, where accessibility differed between CMV responders (**Supplementary Table S4**). Most of these regions (34 out of 35) were more open in donors of high CMV responders. For example, 4 of those differentially accessible sites were annotated to the *NALCN* gene that codes nonselective cation channel (29).

### T-box motifs are specific for CD8^+^ Temra cells

**Figure 6:**
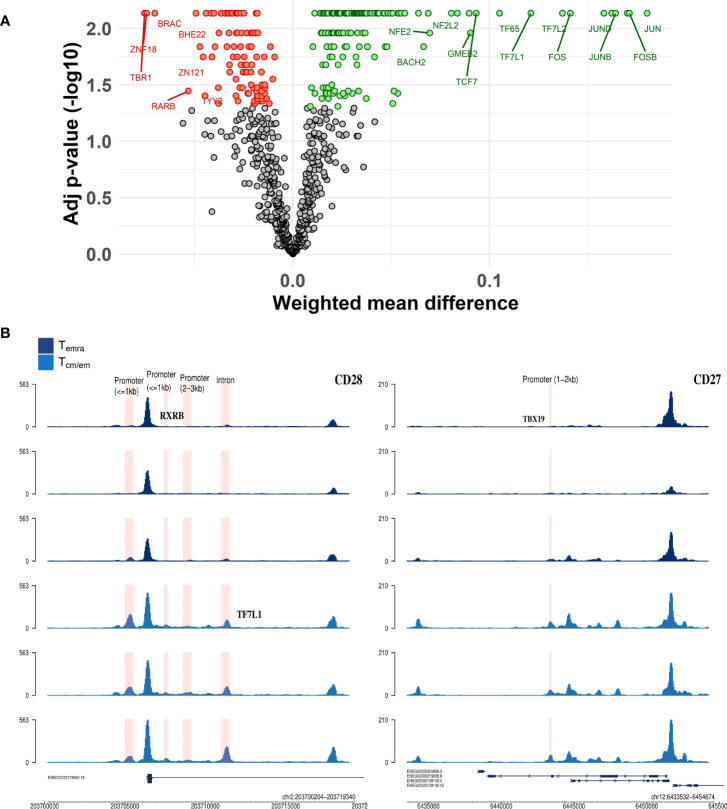
CD8^+^ Temra and Tcm/em cell specific transcription factors. **(A)** Volcano plot of transcription factors that had differential activity between Temra and Tcm/em cells. TFs that were more active in Temra cells are colored red, more active in Tcm/em are colored green and TFs that had adjusted p-value >0.05 are colored grey. The volcano plot shows weighted mean difference (x-axis) versus -log10 adjusted p-value (y-axis). **(B)** Genome tracks showing transcription factor binding sites inside regions/genes that were differentially accessible between Temra and Tcm/em cells. Tcm/em, central and effector memory T cells; Temra, terminally differentiated effector memory T cells.

We used a program called diffTF (30) to calculate differential transcription factor (TF) accessibility between CD8^+^ Tcm/em and Temra cell samples. Chromatin peaks in Tcm/em were strongly associated with the cellular activators such as Jun/Fos, NFkappaB, and STAT (**Figure 6A**). In contrast, Temra cells had a higher representation of T-box (TBR1, TBXT, TBX19) and zinc finger (ZNF18, ZNF121, ZNF631) transcription factor motifs. As TBR1, TBXT and TBX19 are not expressed in T cells, the motifs of T-box factors, identified by diffTF likely represent the binding sites of T-bet (TBX21) and EOMES, the two reciprocally acting T-box transcription factors in CD8^+^ T cell differentiation (31). We next searched for top TF (based on their activity) binding sites within the vicinity of 51 genes that overlapped in their transcriptome and epigenome changes. Motifs for TF7L1 (more active in Tcm/em) and TBX19 (more active in Temra) located in the intron of *CD28* gene and promoter of *CD27* gene, respectively (**Figure 6B**). Moreover, in the promoters of *KLF7* and *MAL* genes, there were binding sites for Eomes and TBX21, respectively (**Supplementary Figure S8**). KLF7 overexpression transforms T cells into less differentiated state (32), while MAL is involved in T cell signal transduction (33). Together, our results show that Temra cells have higher activity of T-box transcription factors compared to Tcm/em cells.

### Sorted CCR7^lo^ CD45RA^hi^ Temra cells are heterogenous

**Figure 7:**
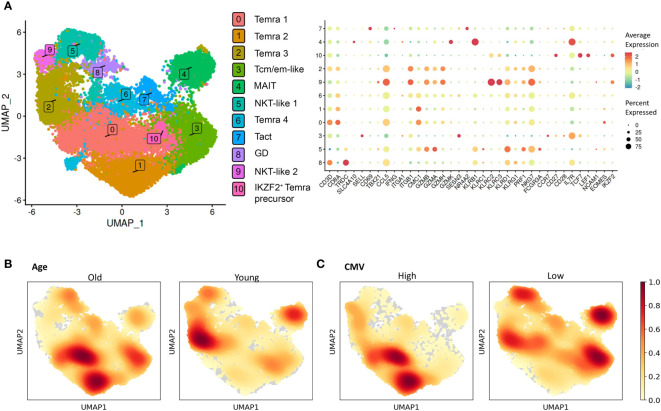
Single-cell analysis of CD8^+^ Temra cells. **(A)** Two dimensional UMAP visualization of the sorted CD8^+^ Temra cells and expression of TOP DEGs (bottom graph). The size of the dot shows the proportion of cells with expression in each cluster. Color of the dot shows average expression of all cells in each cluster. Two dimensional UMAP visualization of the sorted CD8^+^ Temra cells colored by age **(B)** and CMV status **(C)** of the individuals.

Although defined by CCR7 and CD45RA, the late differentiated CD8^+^ T cells are known to be a heterogeneous population (24, 34). For a more detailed analysis of terminally differentiated CD8^+^ T cells, we applied single-cell transcriptome profiling in a cohort of 11 individuals to characterize Temra-related subpopulations and their relation to age and CMV infection. We sorted the CD8^+^ Temra population marked by CCR7^lo^ CD45RA^hi^ from young (n=4) and old (n=7) individuals with high (n=4) and low (n=7) CMV antibody levels and analyzed their single-cell profiles with 10x Genomics technology. After merging and pre-processing the data we identified 10 clusters based on the gene expression patterns, which we annotated by known marker genes (**Figure 7A**; **Supplementary Figure S9**). Single-cell clustering showed cellular heterogeneity and did not directly correspond to the known populations identified in flow cytometry as the differentiation-related marker genes varied and showed intermediate expression among the clusters. This is expected as in contrast to the expression levels in single cell analysis, the flow cytometry applies the splicing difference of CD45 (RA vs RO) as a marker to identify Temra cells. Nevertheless, four cell clusters (0, 1, 2, and 6), including the three largest subpopulations, expressing CD3, CD8A, CCL5, KLRD1 and NKG7 likely corresponded to Temra cells (Temra 1-4). Temra 1, but also 2 and 4, weakly expressed EOMES. We performed a differential expression analysis to identify genes that distinguished these cell subsets (35) (**Supplementary Table S5** **).** The most distinguishing gene in Temra 1 and 2 was CMC1, which was also highly expressed in the transcriptome of Temra population (**Supplementary Table S1**). Temra 3 was negative for CMC1 but had high expression of granzymes. Clusters 5 (FCGR3A^+^, KLRD1^+^, PRF1^+^) and 9 (KLRC2^+^, KLRC3^+^, NKG7^+^) were assigned to NKT-like populations, as they expressed low levels of CD3, CD8A, CD8B, and TYROBP (DAP12), but also shared granzyme and perforin gene markers with CD8^+^ Temra 3 cells. We stained the clusters with 18 Temra and NK cell markers confirming the higher expression of cytotoxic and lack of IL7R genes in Temra and NKT-like cells (**Supplementary Figure S10**). In addition to Temra and NKT-like cells, cluster 3 expressed genes *SELL*, *CCR7* and *CD28*, which we named Tcm/em-like. This population was positive for IL7R that was also highly expressed in MAIT cells, which were represented by a distinct KLRB1^+^ population (cluster 4, SLC4A10^+^). Cluster 7 expressed early activation genes CD69 and NR4A2, representing activated T cells (Tact). Among the sorted cells, we also found gamma-delta T cells (cluster 8, TRDC^+^, TRGC1^+^) and a small population corresponding to IKZF2 Temra precursors (cluster 10, CD27^+^, LEF1^+^, TCF1^+^, IKZF2^+^) (**Figure 7A**, **Supplementary Figure S9**). Our analysis of CD3^+^, CD8^+^, CCR7^lo^ CD45RA^hi^ flow cytometry sorted cells show a heterogeneity of these cells consisting of several CD8^+^ Temra and NKT subset populations with overlapping marker characteristics as well as related cells such as memory cells, MAIT and gamma-delta T cells that might come along with the sorting gating.

### Abundance of single-cell clusters among age and CMV groups

We next compared cell abundance across clusters to determine whether age and CMV altered the subpopulation distributions within the sorted CCR7^lo^ CD45RA^hi^ CD8^+^ T cells. For this, we calculated the density of cells from different conditions and overlaid it on the UMAP plot (**Figure 7B–C**). We also compared each cluster separately between the age and CMV groups (**Supplementary Figures S11**, **12**). However, the studied individuals had substantial variation in their subset proportions and most of the differences were not statistically significant. Among main clusters, we observed altered frequencies of Temra 1, 2 and 3, and MAIT cells in aged individuals. CMC1^+^ Temra 1 and 2 were overrepresented among older whereas Temra 3 population was more enriched in young individuals. We also found statistically significant decrease of IL7R-expressing Tcm/em-like and MAIT cells in CMV^hi^ individuals, however, this association was not present in comparison between old and young individuals. Our pseudobulk analysis of differentially expressed genes in age and CMV group did not reveal substantial differences, suggesting that the cell subsets and not individual genes are altered among the sorted cell population. The results suggest that Temra populations in old and CMV^hi^ individuals are altered and shifted towards CMC1^+^ Temra population whereas younger individuals tend to have more Tcm/em-like and MAIT cells.

## Discussion

The composition of CD8^+^ T cell subsets changes with age and is influenced by the chronic CMV infection, resulting in the accumulation of late-differentiated Temra cells. To further characterize the immunosenescent CD8^+^ Temra population we studied their transcriptomic profiles and compared the gene expression to CD8^+^ T cell subpopulations from CMV^hi^ old individuals. We correlated the transcriptomic findings to chromatin accessibility and identified the regulatory regions of transcription factors active in Tcm/em and Temra cells. We also studied the sorted Temra cell population’s heterogeneity by single-cell approach and its variation in relation to age and CMV.

As expected, old individuals had fewer Tna cells than late differentiated cells as naïve cells gradually differentiate into memory and Temra cells (3). Several studies have reported differences in transcriptome of CD8^+^ T cell subsets, although not all have included Temra cells (17, 36, 37). Accordingly, we found that Tcm/em and Temra were transcriptionally relatively similar but more different from Tna cells. Furthermore, Temra cells had a high expression of NK cell receptor-related genes, as reported earlier (34, 37, 38), whereas Tcm/em cells retained the expression of homing chemokines *CCR4* and *CCR7*, and *SESN3* regulating the AMPK-mTORC1 pathway. Although the Sestrin family members SESN1 and SESN2 have been shown to induce the NK phenotype in CD8^+^ T cells (34), our transcriptome analysis showed decreased expression of *SESN3* in the Temra population and no differential expression of *SESN1* and *SESN2*. Low expression of SESN3, CD27 and CD28 in Temra cells was previously shown by Willinger et al. (39).

We did not find the correlation between Tcmv and Temra or Tcm/em cells in studied individuals. The reason for this might be that the Tcmv cells are not exclusively Temra nor Tcm/em cells as the flow cytometry profiling showed them rather a mixed population by CCR7 and CD45RA markers. Alternatively, the lack of correlation might be explained by other infections that the donors have undergone, decreased responsiveness of Temra cells to antigens, or our small cohort size. Our gene expression data suggested that in average the CD8^+^ Tcmv cells are transcriptionally similar to Tem/cm and Temra cells but different from Tna cells, which also supports our finding that Tcmv cells are a mixed population of memory and Temra cells. Considering the mixed population of CD8^+^ Tcmv cells, it is conceivable that recurrent CMV reactivations stimulate old but also expands existing CMV-specific Tcm/em repertoire, which eventually differentiate into Temra cells, finally lose their TCR-responsiveness and accumulate in old individuals. One limitation of our analysis that the transcriptome differences between CD8^+^ T cell subsets could be affected by their stimulation status. In this respect, the stimulation with unrelated antigens or the transcriptomic analysis of CMV-stimulated CD8^+^ T cell subsets would need further studies.

Compared to Tcm/em cells, our ATAC-seq analysis showed remarkably fewer open chromatin regions in Temra cells (127 vs 1322) suggesting relatively closed chromatin in the Temra population. Amongst the open regions in Temra cells, one of the top open chromatin regions was associated with *ADAM28*, which has been reported to have a higher expression in Temra cells by Callender et al. (38). Our ATAC-seq analysis identified 51 gene regions, which overlapped in their chromatin accessibility and transcription, many with functional roles in T cells. These included co-stimulatory molecules *CD28*, *CD27* and *ICOS*, chemokine receptors *CCR4*, *CXCR5*, and *CCR8* but also NK cell-related *NCR1*, *KLRG1*, and *PALLD* gene encoding cell adhesion cytoskeletal protein. Our study suggests the downregulation of costimulatory molecules CD28 and CD27 in Temra cells is mediated via epigenetic regulation. The lack of CD28 and CD27 expression in T cells is often considered as a characteristic of senescent T cells as it renders T cell unresponsive to antigen stimulation. CD28-negative CD4^+^ T cells have a changed expression and DNA methylation landscape within ca 300 genes involved in T cell cytotoxicity, cytokine/chemokine signaling and the TCR signaling pathway (40). In Temra cells we found higher expression and more accessible chromatin in the promoter region of *NCR1* gene, which encoded NKp46 protein is a NK cell activating cytotoxicity receptor, supporting the view that Temra cells gain more NK-like phenotype and cytotoxicity functions. NKp46 signaling elicit IFN-γ secretion and tumor cell cytotoxicity in NK cells and NKp46 deficient mice are susceptible to various viruses, including influenza virus and murine CMV (41, 42). Thus, the upregulation NCR1 in Temras might provide enhanced protection against the CMV reactivations. Another gene activated and epigenetically regulated in Temra cells was *PALLD*, a component of actin-containing microfilaments that is involved in controlling cell shape and adhesion (43). Whether PALLD functions in Temra cell motility or tissue migration remains to be studied. Thus, CD8^+^ Temra cells have several changes in gene expression and chromatin accessibility when compared to Tcm/em cells, however, their causative relation to Temra cell functional alterations is yet lacking.

We also studied the key transcription factors associated with chromatin accessibility in Tcm/em and Temra cells. The chromatin peaks in Tcm/em cells are associated with multiple activating transcription factors including TCF1, JUN, FOS and STAT and NFkappaB/REL family members. This is in accordance with previous studies as TCF1 represses the development of terminal KLRG1^hi^ effectors (44). TCF1-low cells were also more enriched in effector cells expressing CD57 and showed poorer maintenance of CD27 (45). In contrast, Temra population was associated with T box transcription factors, likely corresponding to the EOMES and TBX21 (T-bet) binding sites in these loci. EOMES (also known as TBR2), T-bet (TBX21), and several other T-box sub-family members share the common DNA-binding consensus site AGGTGTGAAT. Therefore, we considered the real transcription factors in this analysis to be EOMES and T-bet belonging to T-box family. Also, in a recent ATAC-seq analysis on CD8^+^ T cells by Rose et al. (20), T-box members EOMES and TBX21 motifs were associated with chromatin accessibility in memory T cell subpopulations. KIR^+^NKG2A^+^EOMES^+^ CD8^+^ T cell proportions increase with age, have high senescence marker expression, and show a higher degree of differentiation than KIR^+^NKG2A^+^EOMES^-^ CD8^+^ T cells (46). Moreover, we detected high activity of several zinc finger transcription factors (ZFN18, ZFN121) in Temra cells. Similar results were obtained by Giles et al, who constructed human T cell atlas of 14 different CD8^+^ T cell subsets (37).

The senescent CD8^+^ Temra population has been shown to be transcriptionally heterogenic (38). For example, Lu et al. identified 3 Tem and 2 Temra subpopulations among CD8^+^ T cells. Here, we aimed to see the heterogeneity specifically within the CD8^+^CCR7^lo^CD45RA^hi^ sorted cells and demonstrated altogether 10 different cell populations that are comprised in this flow-sorted population. We considered the three largest clusters (0, 1 and 2) and a cluster 6 as Temra cells. These clusters expressed *NKG7*, *GZMH*, *KLRD1*, *CMC1* genes and are likely TCR-responsive because of their CD3 and CD8 expression. Temra 1 and 2 were increased in old individuals and had a distinct expression of CMC1. CMC1 functions in mitochondria, where it works as a chaperon for the cytochrome c oxidase complex that is important in the final steps of electron transfer and ATP production (47). We also noted a high expression of CMC1 in Temra transcriptome, suggesting that CMC1 might have a role in the late differentiation of CD8^+^ T cells. CMC1^+^ Temra subset enrichment in old individuals is supported by findings by Dong et al. showing increased CMC1^+^ and GZMB^+^ cytotoxic T cell clusters in centenarian PBMCs (48). CMC1 expression, however, is ubiquitous and its high expression in Temra cells remains to be studied, though it might indicate the need to stabilize the energy production in mitochondria.

In contrast, Temra 3 population was positive for GZMB and GZMH and might correspond to GZMB^+^ cytotoxic cells reported by Dong and colleagues. GZMB and GZMH were also markers for 2 Temra populations described by Lu et al. (49). A recent extensive single-cell analysis of human PBMCs reported two GZMB^+^ cell clusters among late differentiated CD8^+^ T cells, referred to as GZMB^+^ Tem and Temra (50). In this study, the late differentiated CD8^+^ T cells were not changed between old and young individuals, whereas they found an age-related increase in GZMK^+^ Tem, which were CD45RA^-^ and probably fell outside of our gating strategy. Interestingly, as opposed to CMC1^+^ Temra cells, we found older persons to have fewer Temra 3 cells. As this finding was statistically insignificant because of our small study group, the association of more cytotoxic Temra 3 with younger individuals would need confirmation in a larger cohort and by functional cytotoxicity assays.

We also found two clusters that we named NKT-like cells as they had lower expression of CD3 and CD8. The NKT-like cells expressed cytotoxic enzymes GZMB and GZMA, and NKG7 that regulates the cytotoxic granule exocytosis. It is likely that the NKT-like clusters, having a high resemblance with Temra in their NK cell markers but low CD8^+^ expression, may represent the transition of CD8^+^ Temra to innate NK-like cells with low TCR signaling and high cytotoxic activity (34). However, we did not find increased NKT-cell populations in old individuals, which would have been expected when considering the age-related accumulation of NKT-like cells.

Interestingly, we saw a significant decrease in Tcm/em-like and MAIT populations in CMV^hi^ individuals. As we found that these populations express high levels of IL7R, the result suggests decreased IL7R expression in CMV^hi^ hosts. The higher number of CMV-specific CD8^+^ T cells has been previously associated with low expression of IL7R on blood memory T cells (51). It was proposed that persistent CMV infection results in slower recovery of IL7R on circulating CD8^+^ T cells. In addition, our results suggest that persistent CMV infection also causes a decrease in IL7R^+^ CD8^+^ T cell populations. Although speculative, it may hint that MAIT cells are involved in T-cell response to CMV. Indeed, it has been previously noted that CMV seropositive donors present lower numbers of circulating MAIT cells (52).

In conclusion, our findings demonstrate that CD8^+^ Temra cells are epigenetically more closed and have higher T-box transcription factor activity than less differentiated memory T cells. Furthermore, CD8^+^ Temra cells sorted by standard flow cytometry CR7^lo^CD45RA^hi^ markers constitute a heterogeneous subset of Temra and NKT-like cells. The heterogeneity of the CD8^+^CCR7^lo^CD45RA^hi^ population in peripheral blood is shaped by age and CMV infection, but the phenotype of this cell pool could also be dynamic and responsive to other cues, such as the activation by IL-7 or depend on locations in other tissues. Aside from the classical flow cytometry division of CD8^+^ T cells by CCR7 and CD45 markers, recent single-cell studies have revealed the growing heterogeneity in late differentiated CD8^+^ T cells with several subtypes and gene-specific markers. These newly revealed features of the CD8^+^ T cells, also found by our study, suggest the CCR7 and CD45 markers represent the characteristics of a cell state or cell plasticity rather than cell type and call for a new classification of the late differentiated CD8^+^ T cells.

## Materials and methods

### Study participants and cells

All study participants (**Table 1**) were recruited from Tartu University Hospital from Internal medicine or Dermatology clinics and signed the informed consent. The study was conducted according to the Declaration of Helsinki and approved by the Ethics Review Committee of Human Research of the University of Tartu according to permissions no 272/T-12, 275M-17, and 368/M-4. As the study focused on aging, all participants were 65 years or older. Whole blood samples from donors were collected on several occasions during the routine medical appointments. PBMC-s were isolated by Ficoll-Paque density gradient centrifugation method and stored using CTL-Cryo ABC Media Kit (CTL) in a −150°C freezer. All study participants were studied for their CMV antibodies using luciferase immunoprecipitation system (LIPS) assay as reported in (22).

### CMV peptide stimulation

PBMC-s were thawed in X-Vivo 15 cell medium (Lonza). After washing, mix of diluted costimulants [anti-CD28 (BD) and anti-49d (BD)], pure CD40 antibody (for blocking CD40-CD154 binding; Miltenyi Biotec) and also CMV pp65 peptide pool (1 μg of each peptide per ml, PepTivator, Miltenyi Biotec) were added to each sample. As a negative control, cells were incubated only with costimulants and CD40. PBMCs were stimulated for 16-24h in CO2 enriched thermostat at 37°C.

### Fluorescence-activated cell sorting

2mM EDTA-PBS solution (to detach clumped cells) was added to the stimulated PBMC-s, followed by incubation at 37°C for 10 minutes. After washing, diluted FcR Blocking Reagent (Miltenyi Biotec) and antibody mix were added to each sample and incubated for 30 minutes at 4°C. Antibodies used for sorting were as follows: CD137 PE (Miltenyi Biotec), CD154 BV421 (BioLegend), CD3 AF488 (BioLegend), CD4 AF700 (BioLegend), CD8-BV605 (BioLegend), CD45RA PE-Cy7 (BioLegend) and CCR7 PE-Dazzle (BioLegend; **Supplementary Table S6**). 7-AAD (Miltenyi Biotec) was added to the samples to exclude nonviable cells. Samples were sorted with MA900 Multi-Application Cell Sorter (Sony Biotechnology; gating strategy shown in **Supplementary Figures S3**, **S13A**) and were collected at 4°C into collections tubes containing TRIzol reagent (for transcriptome analysis; Thermo Fisher Scientific) or pre-coated with 4% BSA (for epigenome analysis). Publication-grade FACS plots were generated with the help of FCS Expess 7 (*De Novo* Software).

### RNA isolation, microarray analysis and qPCR

RNA was isolated with RNeasy Micro kit (Qiagen) and the concentration and quality of RNA was measured with NanoDrop (Thermo Fisher Scientific) and Bioanalyzer (Agilent) using RNA 6000 Pico kit (Agilent), respectively. Samples that had RIN (RNA integrity number) ≥ 6.6 were deemed suitable for further analysis. Reverse transcription, DNA labeling, and hybridization of the labeled target to the microarray were done using GeneChip 3’ IVT Pico Kit (Thermo Fisher Scientific) and analyzed by the GeneChip Clariom S Human HT platform (Thermo Fisher Scientific). Microarray plates were read on the GeneTitan (Thermo Fisher Scientific) instrument and analyzed with the TAC 4.0 software. After the analysis, we used a threshold of at least a two-fold expression change (FDR-adjusted p-value < 0.05) to define the differentially expressed genes among CD8^+^ T cell subsets. Gene set enrichment analysis (GSEA) was performed using gProfiler webtool or Genekitr R package. For each sample, 11 μl of total RNA was used as a template for cDNA synthesis. Gene expression results were validated by qPCR (ViiA7, Thermo Fisher Scientific) using SYBR Green/ROX qPCR Master Mix (Fermentas). Primers used are shown in **Supplementary Table S7**. The expression of genes of interest was normalized to the housekeeping gene *β2M* expression and analyzed using the comparative Ct method (53). Tna cells were used as reference group.

### Bionformatic analysis of activation-related genes

To see if any differentially expressed genes between Tcmv cells and resting T cell subsets (Tna, Tcm/em, Temra) are truly differentially expressed or are so due to stimulation status of Tcmv cells, we conducted an additional bioinformatic analysis. For that, we compared our list of differentially expressed genes to the list of differentially expressed genes from Rose et al. study (20), who used RNA-seq to compare transcriptomes of stimulated and unstimulated CD8^+^ T cell subsets.

Specifically, we compared

1. Our list of Tcmv vs Temra with unstimulated Temra cells vs stimulated Temra cells from Rose et al. study.
2. Our list of Tcmv vs Tcm/em with unstimulated Tcm cells vs stimulated Tcm cells from Rose et al. study.
3. Our list of Tcmv vs Tcm/em with unstimulated Tem cells vs stimulated Tem cells from Rose et al. study and found the overlapping genes. Then we removed the overlapping genes (Tcm and Tem were combined) from our list of differentially expressed genes and re-ran the analysis.

### ATAC-seq and downstream analysis

Differentially accessible chromatin regions were analyzed by ATAC-seq according to the protocol by Buenrostro et al. (54). Briefly, 50 000 CD8^+^ Temra and Tcm/em cells were isolated by FACS, followed by transposition and DNA purification steps (GeneJET Gel Extraction and DNA Cleanup Micro Kit, Thermo Fisher Scientific). Then the experiment was put on hold and DNA samples were stored in -20°C freezer until all the samples were collected. The correct number of cycles for PCR was calculated with the help of qPCR and additional 5 cycles were added. The quality of libraries was evaluated by Qubit (Thermo Fisher Scientific), and TapeStation (Agilent). All libraries were sequenced on the Illumina NextSeq500 with 75 bp paired-end reads. After sequencing, preprocessing of the data was done using the Galaxy web platform. First, Illumina adapters were trimmed by Cutadapt. In addition, Cutadapt was used to filter out low quality (quality less than 20) and short reads (< 20 bp). Samples were aligned to hg38 with Bowtie2 using *–dovetail* and *–very-sensitive* options. Next, reads that mapped to the mitochondrial genome, had low mapping quality, and didn’t pair properly, were removed. The distribution of fragment lengths (**Supplementary Figure S13B**) and mapped reads (**Supplementary Figure S13C**) was acquired from all ATAC-seq libraries and indicated a good quality. Peak calling was done by Genrich using ATAC-seq mode. DiffBind was used for group comparison (log2 fold change ≥ abs (2); FDR-adjusted p-value < 0.05). Peaks only present in at least two samples were included in the analysis. The consensus peakset was generated gathering all peaks from both cell types. Differentially open regions were annotated to genes using ChipSeeker package.

### Differential transcription factor activity analysis

diffTF (30) was used to calculate differential transcription factor accessibility between CD8^+^ Tcm/em and Temra cells. diffTF uses ATAC-seq and transcriptional data such as RNA-seq or microarray data and classifies TFs into transcriptional activators or repressors by quantifying and correlating TF motif accessibility. diffTF was ran in default mode using 1,000 permutations. Normalized microarray data, peakset with 65,669 peaks (representing the consensus peakset of all samples that overlaps at least 10 different samples) together with recommended and pre-calculated transcription factor data (based on HOCOMOCO v11 motifs + PWMScan, see diffTF documentation) were used as input.

### Single-cell analysis

scRNAseq was carried out in two different reactions consisting of 5 or 6 samples (**Supplementary Figure S14**). Samples were combined and barcoded so that every reaction contained one young CMV^hi^ and CMV^lo^ sample and two (or one) elderly CMV^hi^ and CMV^lo^ samples. For single-cell analyses, previously extracted and frozen PBMCs were thawed using RPMI 1640 media (Corning) supplemented with Penicillin-Streptomycin (Corning) and Fetal Bovine Serum (Gibco). After washing with media, cells were transferred to a cell culture dish for overnight incubation in previously described RPMI 1640+FBS+PS media. The next morning cells were collected and labeled simultaneously with TotalSeq-B Hashtags (BioLegend), CD8 Microbeads (Miltenyi Biotech) and FACS antibodies: CD3 Alexa Fluor 488, CD4 Alexa Fluor 700, CCR7 PE-Dazzle 594 and CD45RA PE-Cy7 (all from BioLegend) in 1% BSA in PBS. CD8^+^ cells were extracted using MS columns and MACS separator (both from Miltenyi Biotech). Cells were washed according to the 10X Genomics protocol CG000149 Rev C. After the washes, Temra cells were sorted using Sony MA900 Cell Sorter (Sony Biotechnology) and counted using Luna FL Automated Cell Counter and Acridine Orange/Propidium Iodide Stain (Logos Biosystems). Approximately 5000 Temra cells from 5-6 individuals with different barcodes were combined into one reaction and loaded onto the Chromium controller and cDNA was generated using Chromium Single Cell 3’ Reagent Kit (v3.1 Chemistry Dual Index) and Chromium Next GEM Chip G Single Cell Kit (both from 10X Genomics) according to manufacturer’s instructions. Gene expression and hashtag libraries were paired-end sequenced on an Illumina NovaSeq 6000 instrument in Novogene.

The raw sequencing data were processed using the 10x Genomics Cell Ranger pipeline (version 6.1.2) with the GRCh38 reference. The scRNA-seq alignment and quantification were carried out on the Tartu University High Performance Computing Center Rocket cluster. The samples were demultiplexed using the HTODemux function from the Seurat package (35). The barcodes having reads for more than one molecular hashtag were designated as doublets and excluded from the downstream processing. Further filtering was applied to remove the cells with less than 1100 and more than 12% of mitochondrial genes. We have observed cell populations that expressed high levels of CD68, which is a monocyte marker. These cells were excluded from further analyses.

The transcriptomes of the retained high-quality cells were further processed according to the Seurat data integration workflow to account for the between-batch technical differences. In brief, the data were normalized using the SCTransform function (version 2). The integration features were selected as the intersection of the top within-batch highly variable genes. Next, the integration was performed as previously described (55). The first 30 principal components were selected for building the UMAP projection and clustering the data. To visualize the cell density in CMV and aging, the data were exported for processing in Scanpy package. Gaussian kernel density estimation was used to calculate the density of cells in a UMAP space (56).

The cell identities were assigned based on a combination of the differentially expressed genes (DE) for each cluster and were identified using the FindAllMarkers function in Seurat (Wilcoxon Rank Sum test). The DE marker genes of each cluster were determined based on the following criteria (1): expressed in more than 10% of the cells within either or both two groups (2); absolute value of the log fold change > 0.25 (3); adjusted p-value < 0.01.

## Data availability statement

The datasets presented in this study can be found in online repositories. The names of the repository/repositories and accession number(s) can be found below: PRJNA1005067 (SRA), GSE241027 (GEO), GSE241493 (GEO).

## Ethics statement

The studies involving humans were approved by the Ethics Review Committee of Human Research of the University of Tartu (permissions no 272/T-12, 275M-17, and 368/M-4). The studies were conducted in accordance with the local legislation and institutional requirements. The participants provided their written informed consent to participate in this study.

## Author contributions

LT: Conceptualization, Investigation, Methodology, Validation, Visualization, Formal analysis, Writing – original draft, Writing – review & editing, Data curation. IF: Formal analysis, Visualization, Data Curation, Writing – review & editing, Writing – original draft. CA: Formal analysis, Software, Methodology, Writing – review & editing, Writing – original draft. JZ: Supervision, Writing – review & editing, Writing – original draft. LTs: Conceptualization, Investigation, Methodology, Supervision, Writing – original draft, Writing – review & editing. KK: Conceptualization, Methodology, Project administration, Supervision, Writing – original draft, Writing – review & editing. PP: Conceptualization, Methodology, Funding acquisition, Project administration, Supervision, Writing – original draft, Writing – review & editing.

## Supplementary material

Supplementary Figure 1 Study design. PBMC-s were isolated from the whole-blood and cryopreserved. After the thawing, re-stimulation with CMV antigen and/or cell-surface marker staining was performed. This was followed by sorting step for three different experiments. For gene expression analysis, 4 CD8^+^ T cell subsets were sorted: naïve T cells (Tna – 24 samples), memory T cells (Tcm/em – 28 samples), Temra cells (27 samples) and CMV-responsive CD8^+^ T cells (Tcmv – 12 samples). After RNA extractions all samples were subjected to the microarray analysis. For epigenome analysis, 2 CD8^+^ T cell subsets were sorted: Tcm/em (18 samples) and Temra (24 samples). Chromatin accessibility was studied using ATAC-seq method. For single-cell study, only CD8^+^ Temra cells were sorted (23 samples). This figure was created with BioRender.com.

Supplementary Figure 2 Selected Gene Ontology (GO) terms for biological process (BP) in the gene set enrichment analysis of differentially expressed genes (DEGs): **(A)** Tcm/em vs Tna **(B)** Temra vs Tna **(C)** Temra vs Tcm/em. Tna, naïve T cells; Tcm/em, central and effector memory T cells; Temra, terminally differentiated effector memory T cells.

Supplementary Figure 3 Gating strategy for sorting CD8^+^ T cell subsets. PBMC-s were sorted with MA900 Multi-Application Cell Sorter. First, lymphocytes were gated using complexity (BSC-A) and cell size (FSC-A). Singlets were gated out from lymphocytes, followed by exclusion of nonviable cells using 7-AAD dye. From live cells, T cells were gated by expression of CD3, which were in turn divided into T cell subsets using CD4 and CD8 surface molecules. CD8^+^ T cells were next divided into Tcmv and resting T cells using activation markers CD154 and CD137. Resting T cells were further gated into Tna cells (CCR7^+^CD45RA^+^), Tcm/em cells (CCR7^+/-^CD45RA^-^) and Temra cells (CCR7^-^CD45RA^+^). Mock-stimulated control sample was used to set the gate for the activated T (CD154^+^CD137^+^) cells.

Supplementary Figure 4 Characterization of Tcmv cells. **(A)** There is no correlation between Tcmv and Temra/Tcm/em. **(B)** Pie chart showing proportions of Tcmv samples belonging to mixed, mostly Tcm/em and mostly Temra subgroups shown in figure C. **(C)** FACS plots from sorting experiment showing the division of CD8^+^ Tcmv cells into Temra and/or Tcm/em subsets using surface markers CCR7 and CD45RA. Tcm/em, central and effector memory T cells; Temra, terminally differentiated effector memory T cells; Tcmv, CMV-responsive CD8^+^ T cells.

Supplementary Figure 5 Differences in transcriptome between Tcmv and resting (Tna, Tcm/em and Temra) CD8^+^ T cell subsets. Top 3 up- and downregulated genes in Tcmv cells in comparison to resting T cell subsets represented as boxplots. * Indicates p-value ≤0.05, ** indicates p-value ≤0.01, *** indicates p-value ≤0.001; Tcmv – CMV-responsive CD8^+^ T cells.

Supplementary Figure 6 Subtraction of activation-related genes. Top 20 up- (top graphs) and downregulated (bottom graphs) genes in Tcmv after removing activation-related genes as described in Materials and methods section. **(A)** Tcmv versus Temra, **(B)** Tcmv versus Tcm/em. Tcm/em, central and effector memory T cells; Temra, terminally differentiated effector memory T cells; Tcmv, CMV-responsive CD8^+^ T cells.

Supplementary Figure 7 Examples of overlapping results between transcriptome and epigenome analyses represented as boxplots. Values are centered and scaled (z-scores). Tcm/em, central and effector memory T cells; Temra, terminally differentiated effector memory T cells.

Supplementary Figure 8 Genome tracks showing transcription factor binding sites inside *MAL* and *KLF7* genes that were differentially accessible between Temra and Tcm/em cells. Tcm/em, central and effector memory T cells; Temra, terminally differentiated effector memory T cells.

Supplementary Figure 9 Heatmap showing expression levels of genes differentially expressed across clusters (columns) in single-cell analysis.

Supplementary Figure 10 Two dimensional UMAP visualization of the sorted CD8^+^ Temra cells colored by the expression of selected marker genes.

Supplementary Figure 11 Boxplots showing the percentage of cells of each CD8^+^ T cell cluster between old and young.

Supplementary Figure 12 Boxplots showing the percentage of cells of each CD8+ T cell cluster between CMV^hi^ and CMV^lo^.

Supplementary Figure 13 Proportions of T cell subsets and quality control of ATAC-seq data. **(A)** Percentage of sorted CD8^+^ T cell subsets. Black dot on the graph represents mean. **(B)** Heatmap summarizing ATAC-seq coverage from one sample. **(C)** Fragment Size Distribution of one sample. Tna, naïve T cells,; Tcm/em, central and effector memory T cells; Temra, terminally differentiated effector memory T cells.

Supplementary Figure 14 Overview of the single-cell study design. For single-cell analyses 4 young and 7 elderly individuals were recruited. After the extraction of PBMCs, cells were stained simultaneously with TotalSeq-B Hashtags, CD8 Microbeads and FACS antibodies. Next, CD8^+^ cells were extracted using MS columns and MACS separator. Temra cells were sorted using Sony MA900 Cell Sorter. 5000 Temra cells from 5-6 individuals with different barcodes were combined into one reaction and loaded onto the Chromium controller. The image was created using BioRender.com.

Supplementary Table 1 Lists of differentially expressed genes between CD8^+^ T cell subsets.

Supplementary Table 2 Lists of differentially expressed genes between Tcmv and resting (Tna, Tcm/em, Temra) CD8^+^ T cell subsets.

Supplementary Table 3 List of differentially accessible regions between CD8^+^ Temra and Tcm/em cells.

Supplementary Table 4 List of differentially accessible regions between high and low CMV responders amongst CD8^+^ Temra cells.

Supplementary Table 5 List of differentially expressed genes between CD8^+^ cell subsets identified by single-cell analysis.

Supplementary Table 6 List of antibodies used in flow cytometry experiments.

Supplementary Table 7 List of primers used in qPCR experiment.
