# Supplementary figures and images for: Cytotoxic CD8+ Temra cells show loss of chromatin accessibility at genes associated with T cell activation
Source: Front Immunol. 2024 Feb 2;15:1285798. doi: 10.3389/fimmu.2024.1285798 (PMC10870784; doi:10.3389/fimmu.2024.1285798)

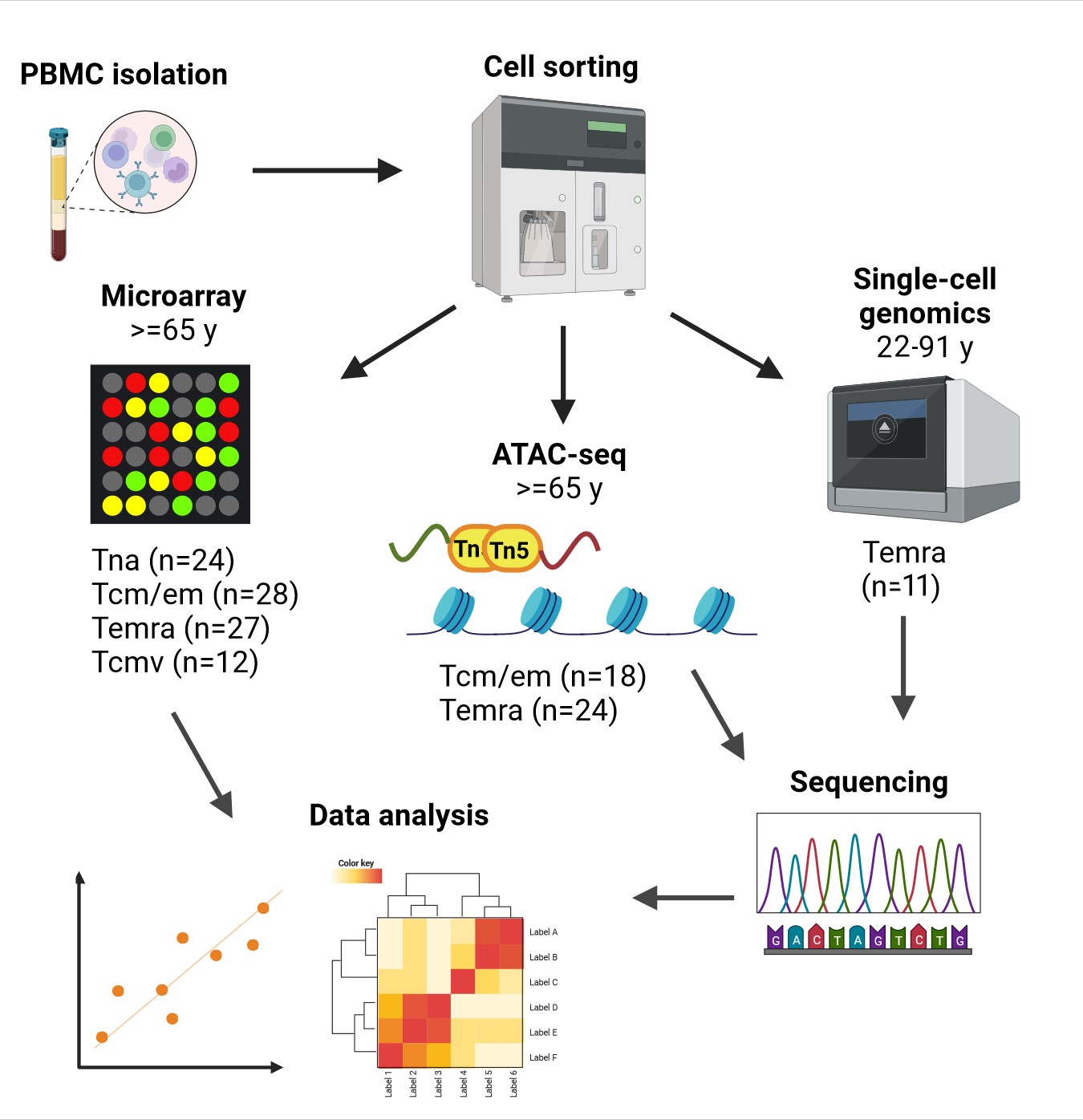

Supplement: Supplementary Figure 1 — Study design. PBMC-s were isolated from the whole-blood and cryopreserved. After the thawing, re-stimulation with CMV antigen and/or cell-surface marker staining was performed. This was followed by sorting step for three different experiments. For gene expression analysis, 4 CD8+ T cell subsets were sorted: naïve T cells (Tna – 24 samples), memory T cells (Tcm/em – 28 samples), Temra cells (27 samples) and CMV-responsive CD8+ T cells (Tcmv – 12 samples). After RNA extractions all samples were subjected to the microarray analysis. For epigenome analysis, 2 CD8+ T cell subsets were sorted: Tcm/em (18 samples) and Temra (24 samples). Chromatin accessibility was studied using ATAC-seq method. For single-cell study, only CD8+ Temra cells were sorted (23 samples). This figure was created with BioRender.com. [file Image_1.jpeg]

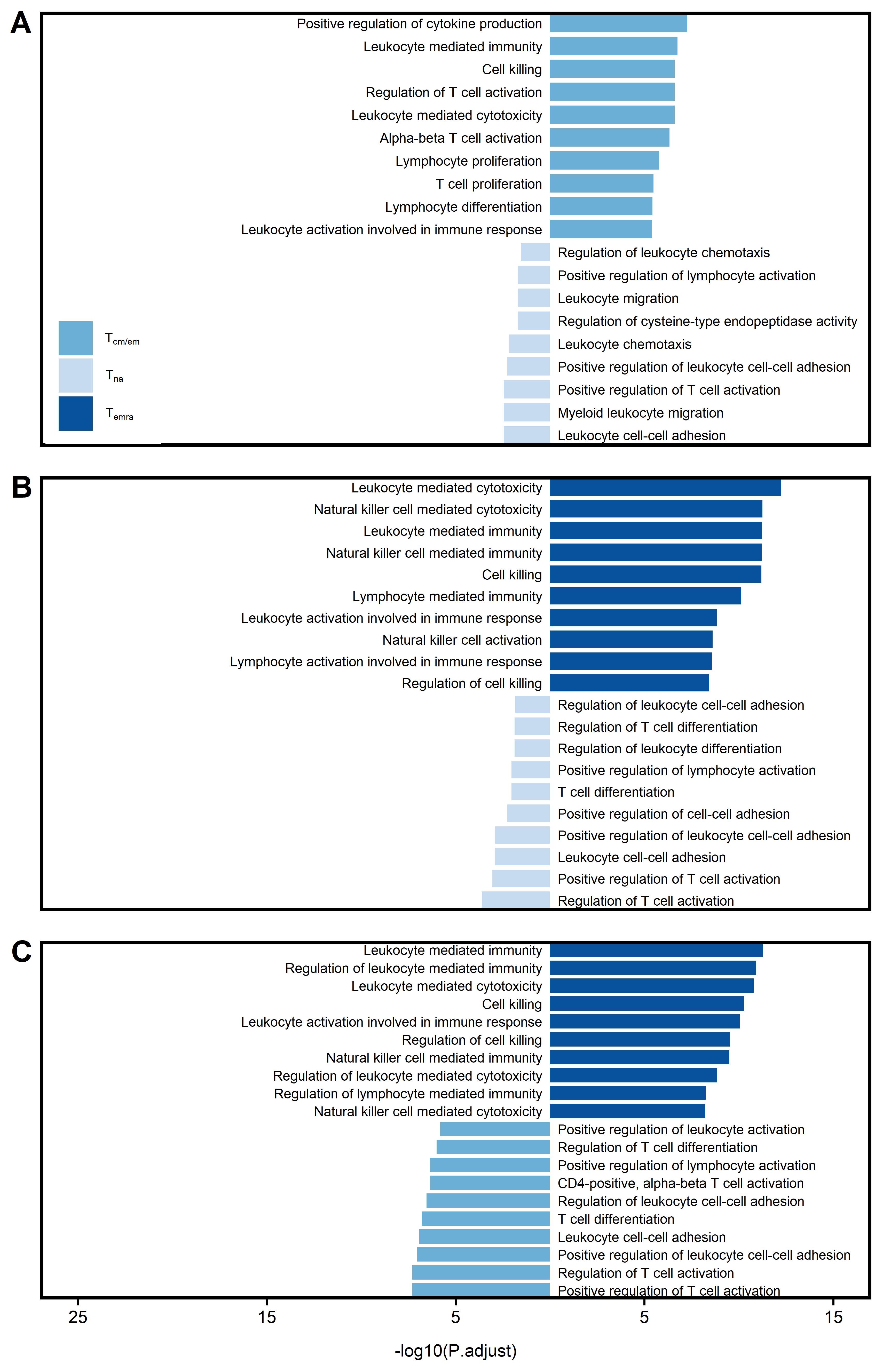

Supplement: Supplementary Figure 2 — Selected Gene Ontology (GO) terms for biological process (BP) in the gene set enrichment analysis of differentially expressed genes (DEGs): (A) Tcm/em vs Tna (B) Temra vs Tna (C) Temra vs Tcm/em. Tna, naïve T cells; Tcm/em, central and effector memory T cells; Temra, terminally differentiated effector memory T cells. [file Image_2.jpeg]

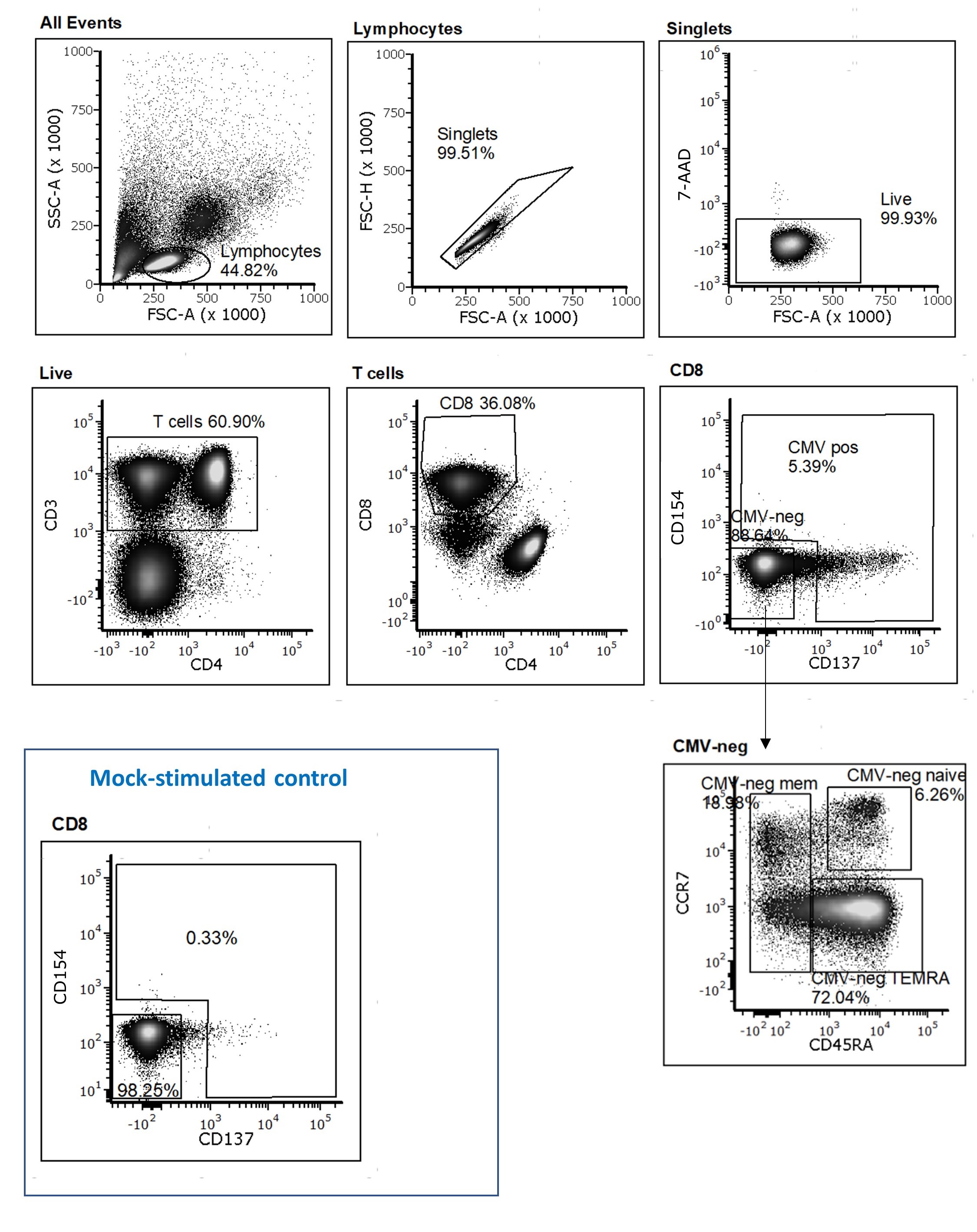

Supplement: Supplementary Figure 3 — Gating strategy for sorting CD8+ T cell subsets. PBMC-s were sorted with MA900 Multi-Application Cell Sorter. First, lymphocytes were gated using complexity (BSC-A) and cell size (FSC-A). Singlets were gated out from lymphocytes, followed by exclusion of nonviable cells using 7-AAD dye. From live cells, T cells were gated by expression of CD3, which were in turn divided into T cell subsets using CD4 and CD8 surface molecules. CD8+ T cells were next divided into Tcmv and resting T cells using activation markers CD154 and CD137. Resting T cells were further gated into Tna cells (CCR7+CD45RA+), Tcm/em cells (CCR7+/-CD45RA-) and Temra cells (CCR7-CD45RA+). Mock-stimulated control sample was used to set the gate for the activated T (CD154+CD137+) cells. [file Image_3.jpeg]

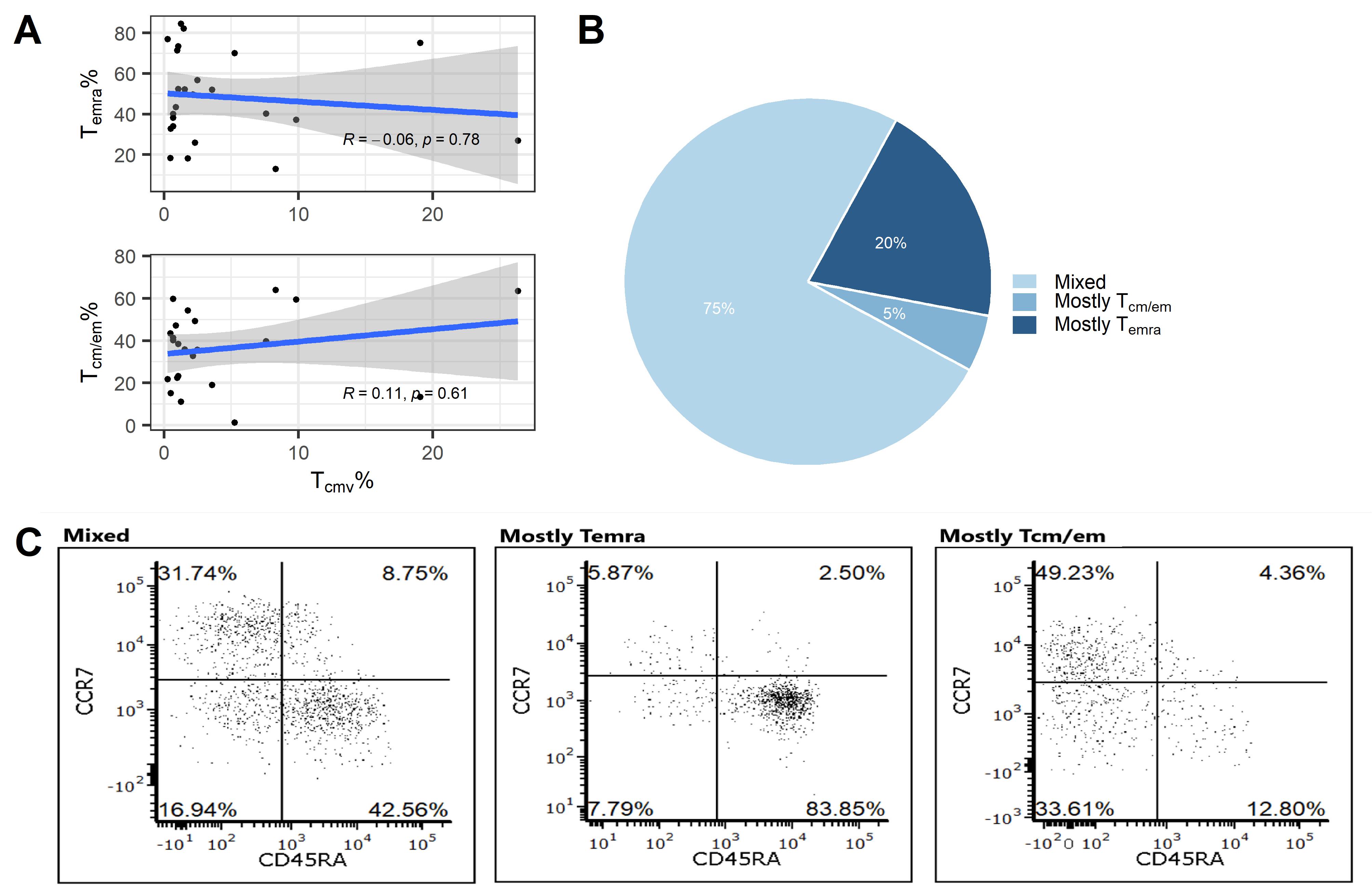

Supplement: Supplementary Figure 4 — Characterization of Tcmv cells. (A) There is no correlation between Tcmv and Temra/Tcm/em. (B) Pie chart showing proportions of Tcmv samples belonging to mixed, mostly Tcm/em and mostly Temra subgroups shown in figure C. (C) FACS plots from sorting experiment showing the division of CD8+ Tcmv cells into Temra and/or Tcm/em subsets using surface markers CCR7 and CD45RA. Tcm/em, central and effector memory T cells; Temra, terminally differentiated effector memory T cells; Tcmv, CMV-responsive CD8+ T cells. [file Image_4.jpeg]

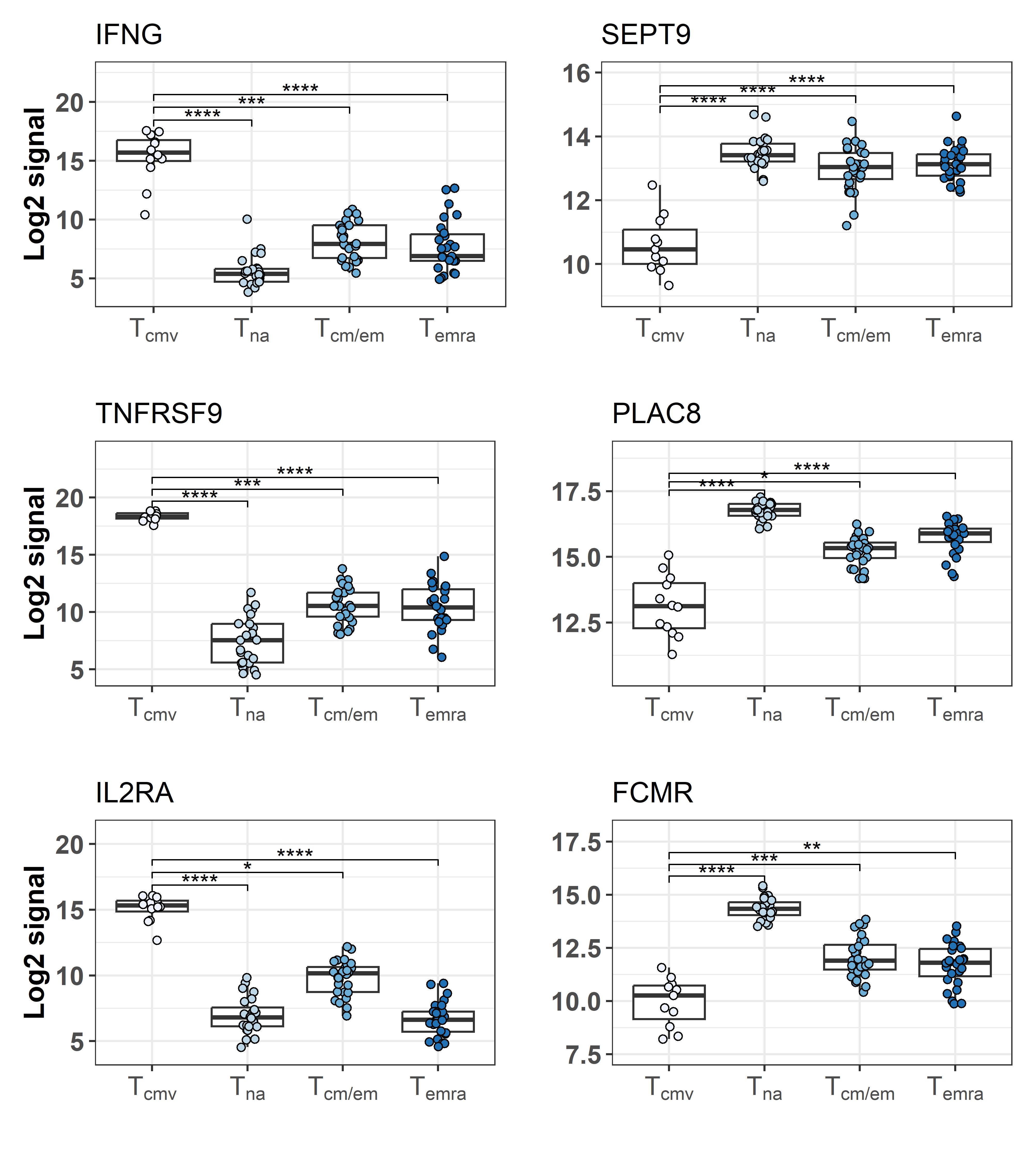

Supplement: Supplementary Figure 5 — Differences in transcriptome between Tcmv and resting (Tna, Tcm/em and Temra) CD8+ T cell subsets. Top 3 up- and downregulated genes in Tcmv cells in comparison to resting T cell subsets represented as boxplots. * Indicates p-value ≤0.05, ** indicates p-value ≤0.01, *** indicates p-value ≤0.001; Tcmv – CMV-responsive CD8+ T cells. [file Image_5.jpeg]

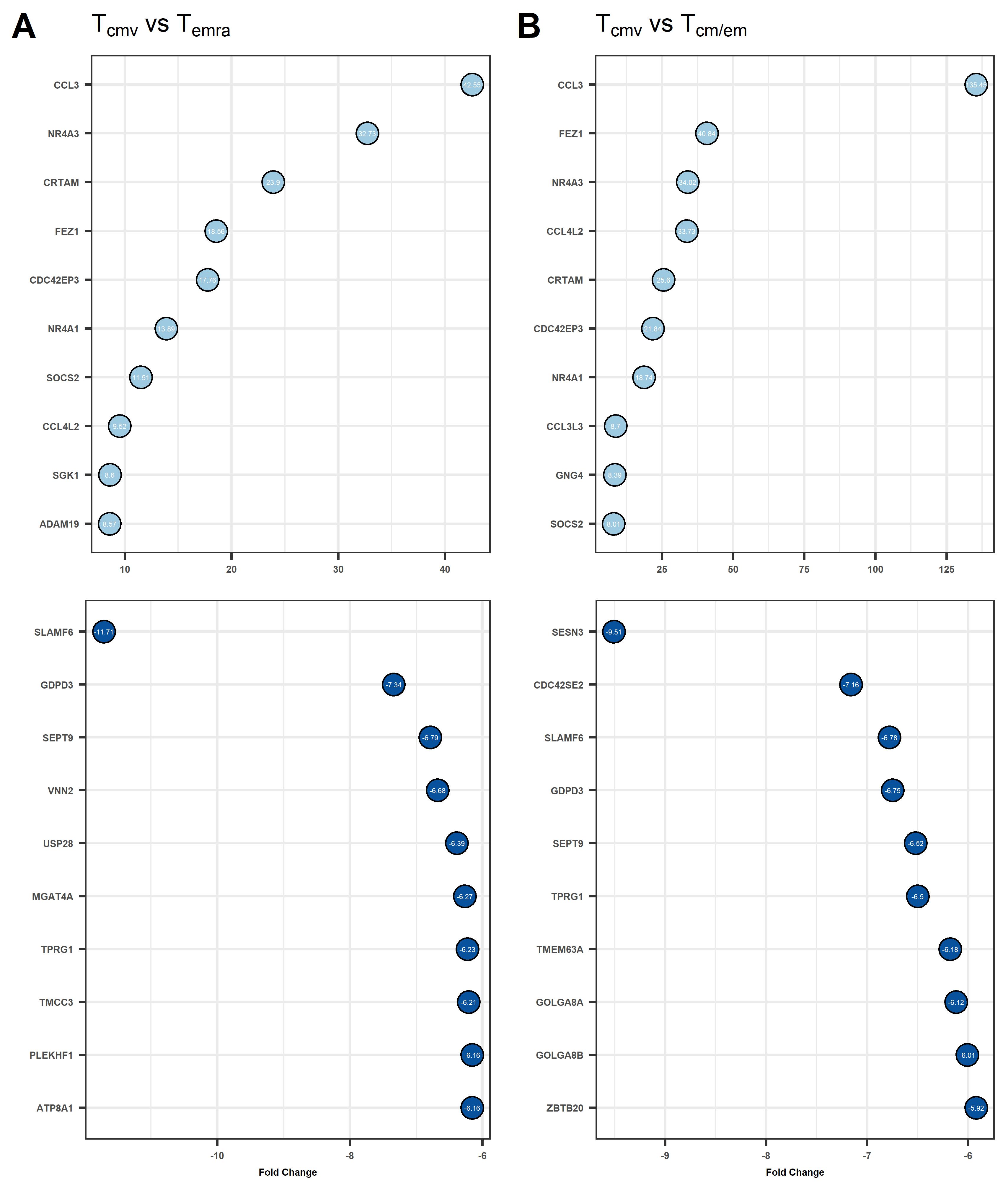

Supplement: Supplementary Figure 6 — Subtraction of activation-related genes. Top 20 up- (top graphs) and downregulated (bottom graphs) genes in Tcmv after removing activation-related genes as described in Materials and methods section. (A) Tcmv versus Temra, (B) Tcmv versus Tcm/em. Tcm/em, central and effector memory T cells; Temra, terminally differentiated effector memory T cells; Tcmv, CMV-responsive CD8+ T cells. [file Image_6.jpeg]

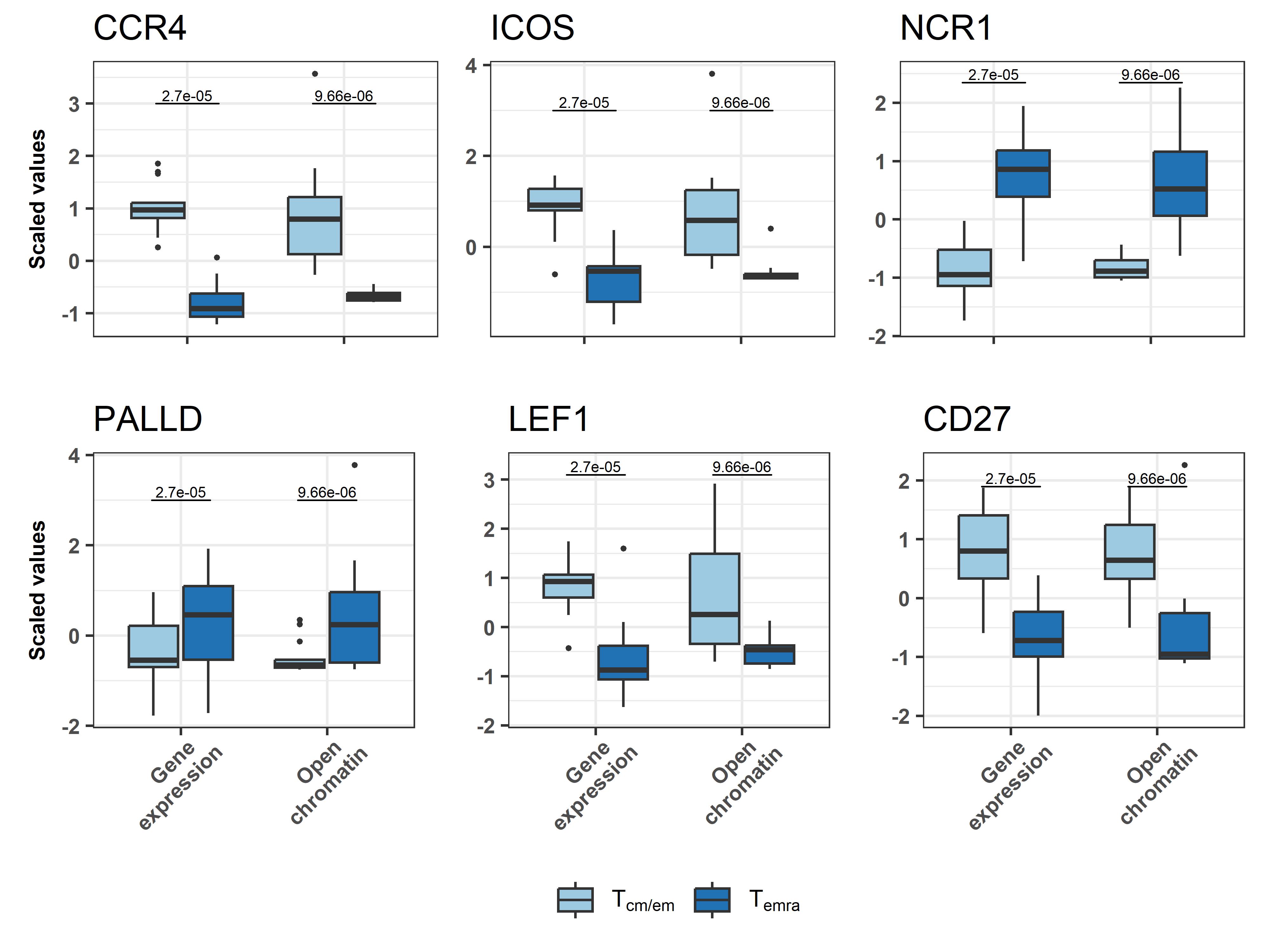

Supplement: Supplementary Figure 7 — Examples of overlapping results between transcriptome and epigenome analyses represented as boxplots. Values are centered and scaled (z-scores). Tcm/em, central and effector memory T cells; Temra, terminally differentiated effector memory T cells. [file Image_7.jpeg]

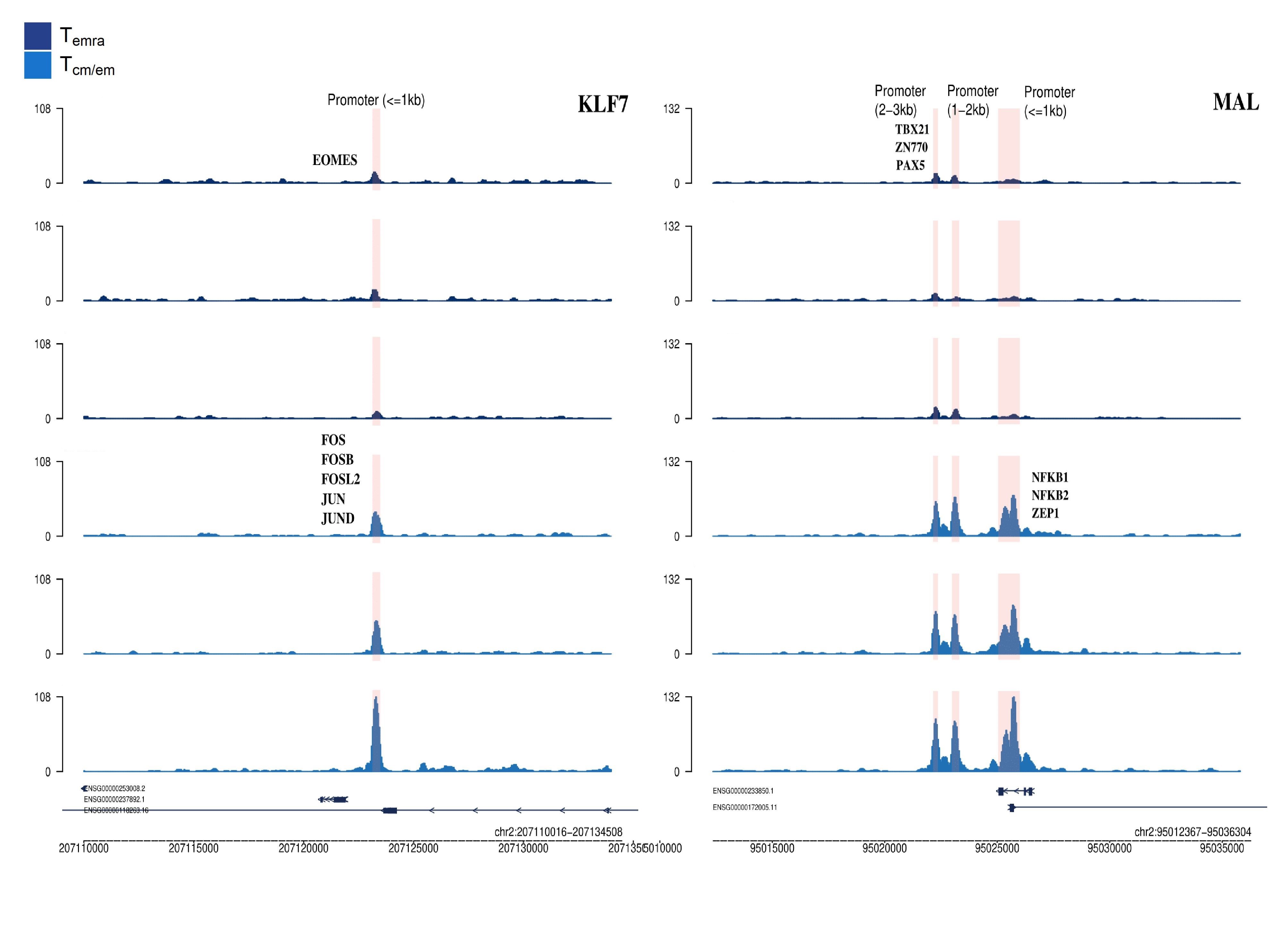

Supplement: Supplementary Figure 8 — Genome tracks showing transcription factor binding sites inside MAL and KLF7 genes that were differentially accessible between Temra and Tcm/em cells. Tcm/em, central and effector memory T cells; Temra, terminally differentiated effector memory T cells. [file Image_8.jpeg]

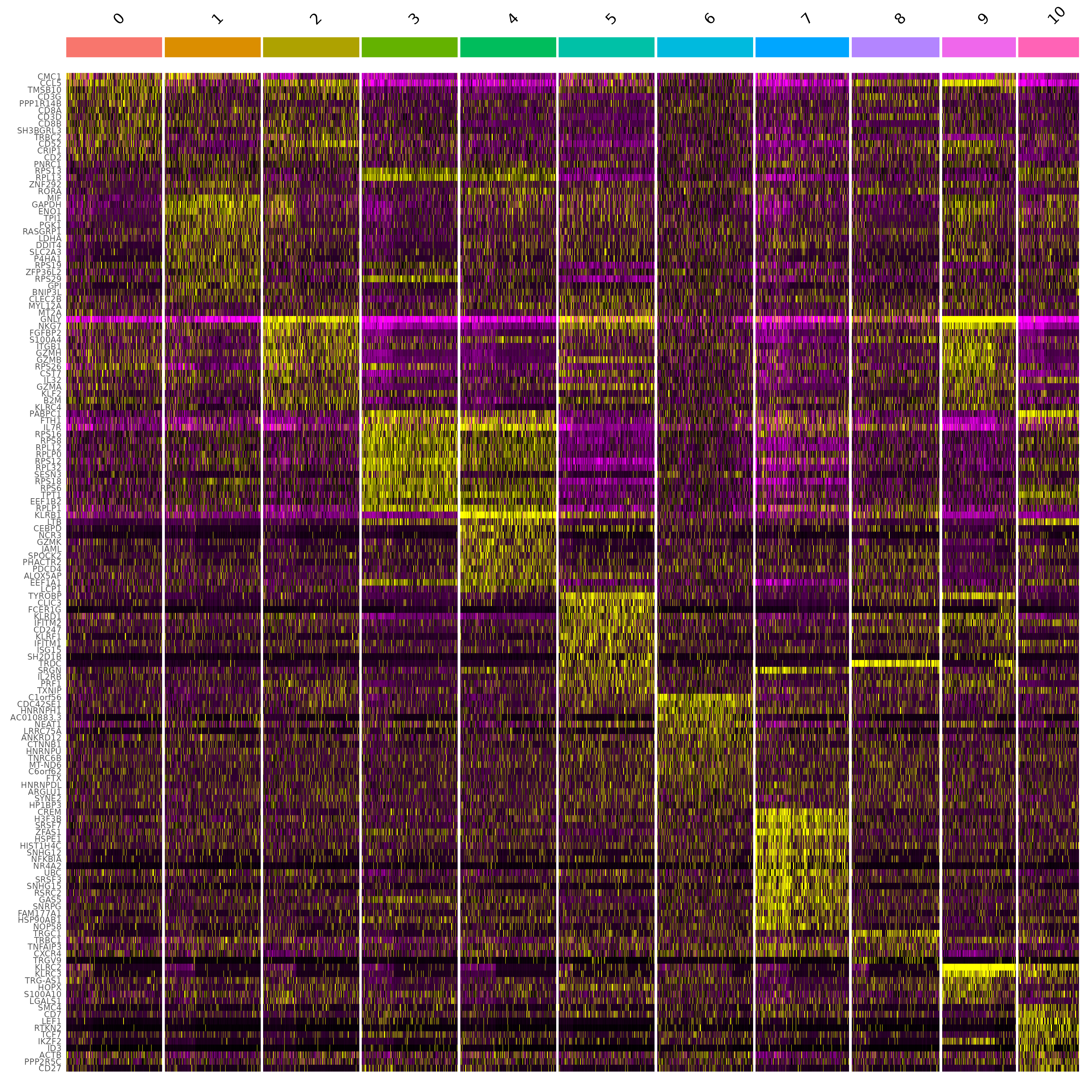

Supplement: Supplementary Figure 9 — Heatmap showing expression levels of genes differentially expressed across clusters (columns) in single-cell analysis. [file Image_9.png]

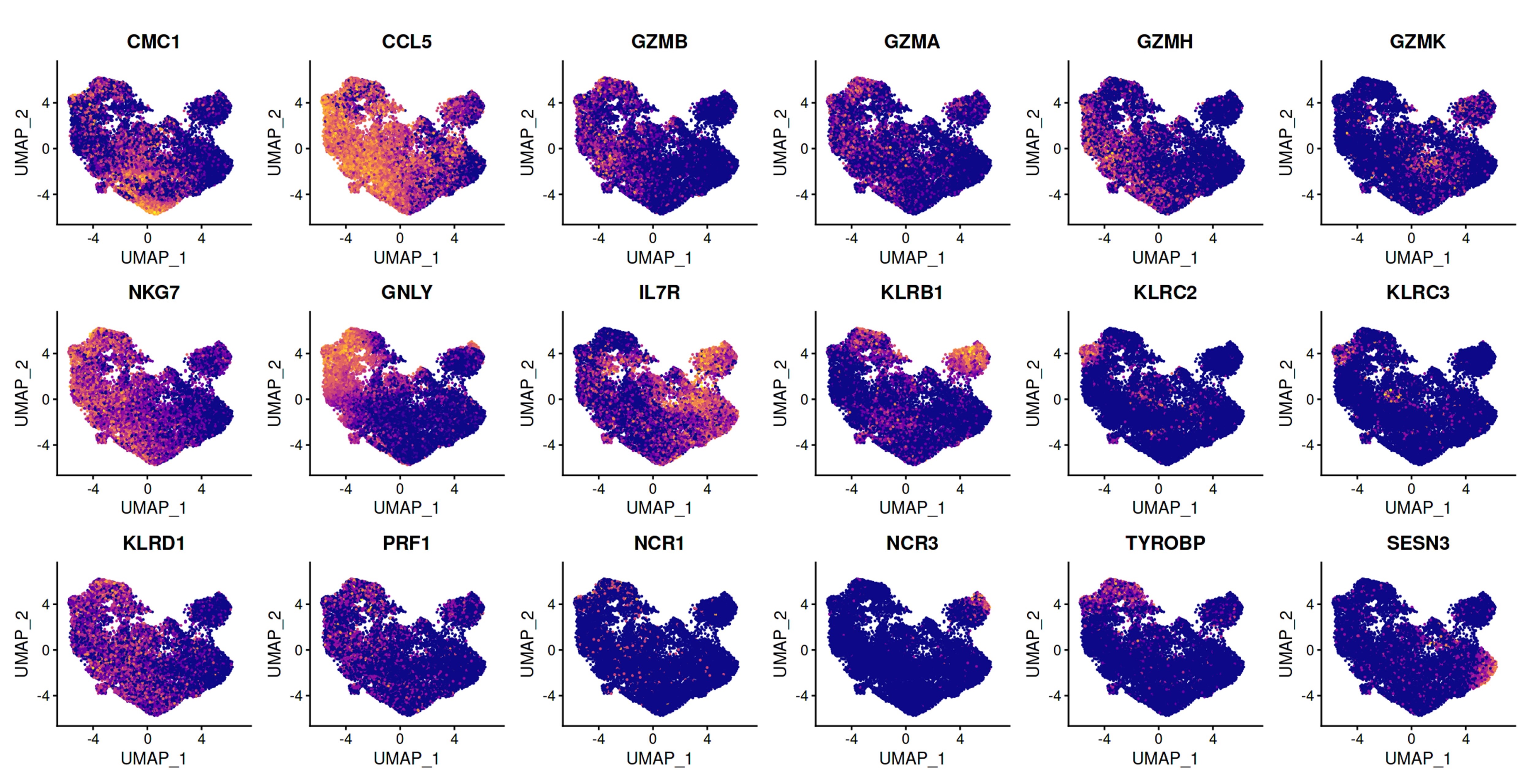

Supplement: Supplementary Figure 10 — Two dimensional UMAP visualization of the sorted CD8+ Temra cells colored by the expression of selected marker genes. [file Image_10.jpeg]

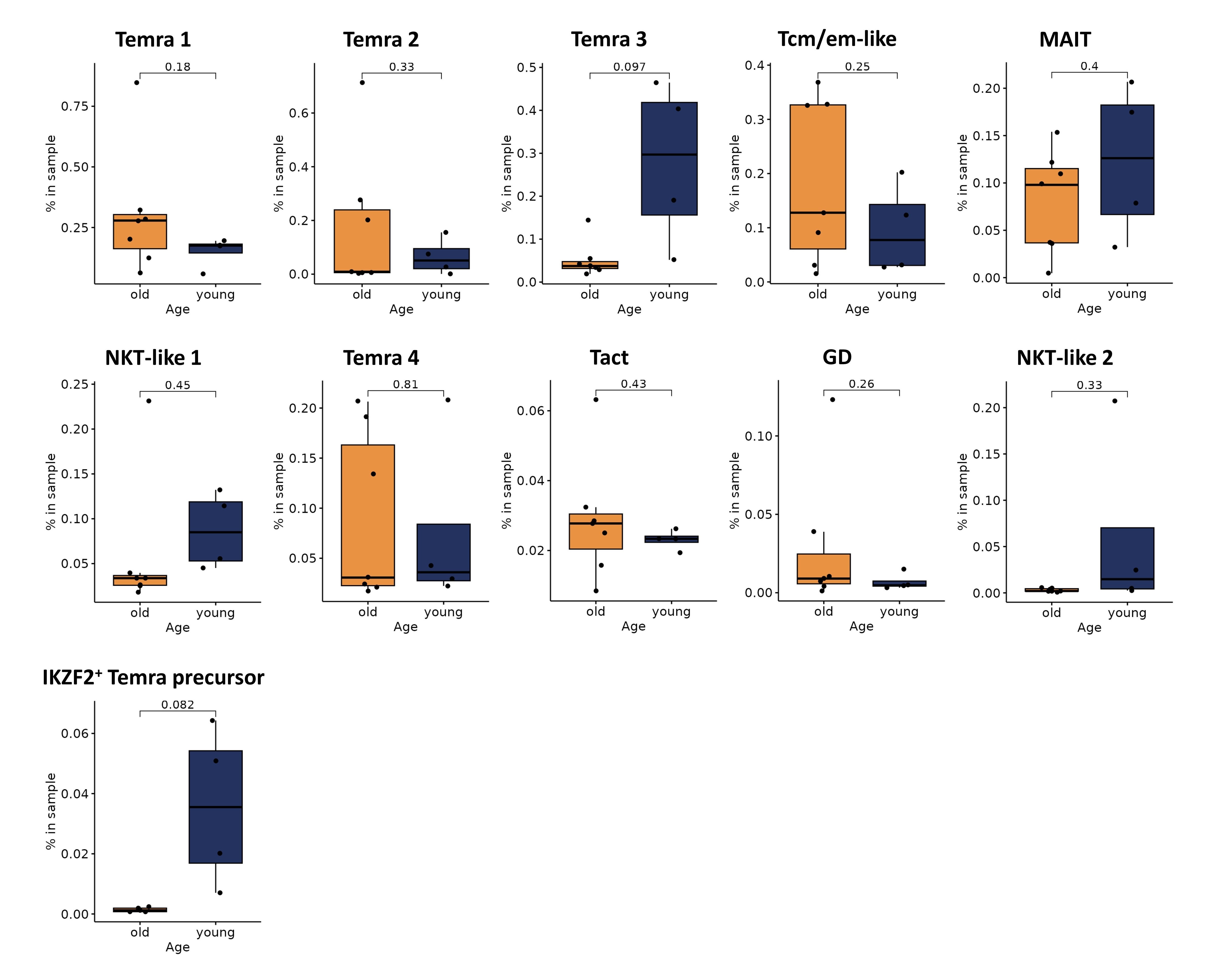

Supplement: Supplementary Figure 11 — Boxplots showing the percentage of cells of each CD8+ T cell cluster between old and young. [file Image_11.jpeg]

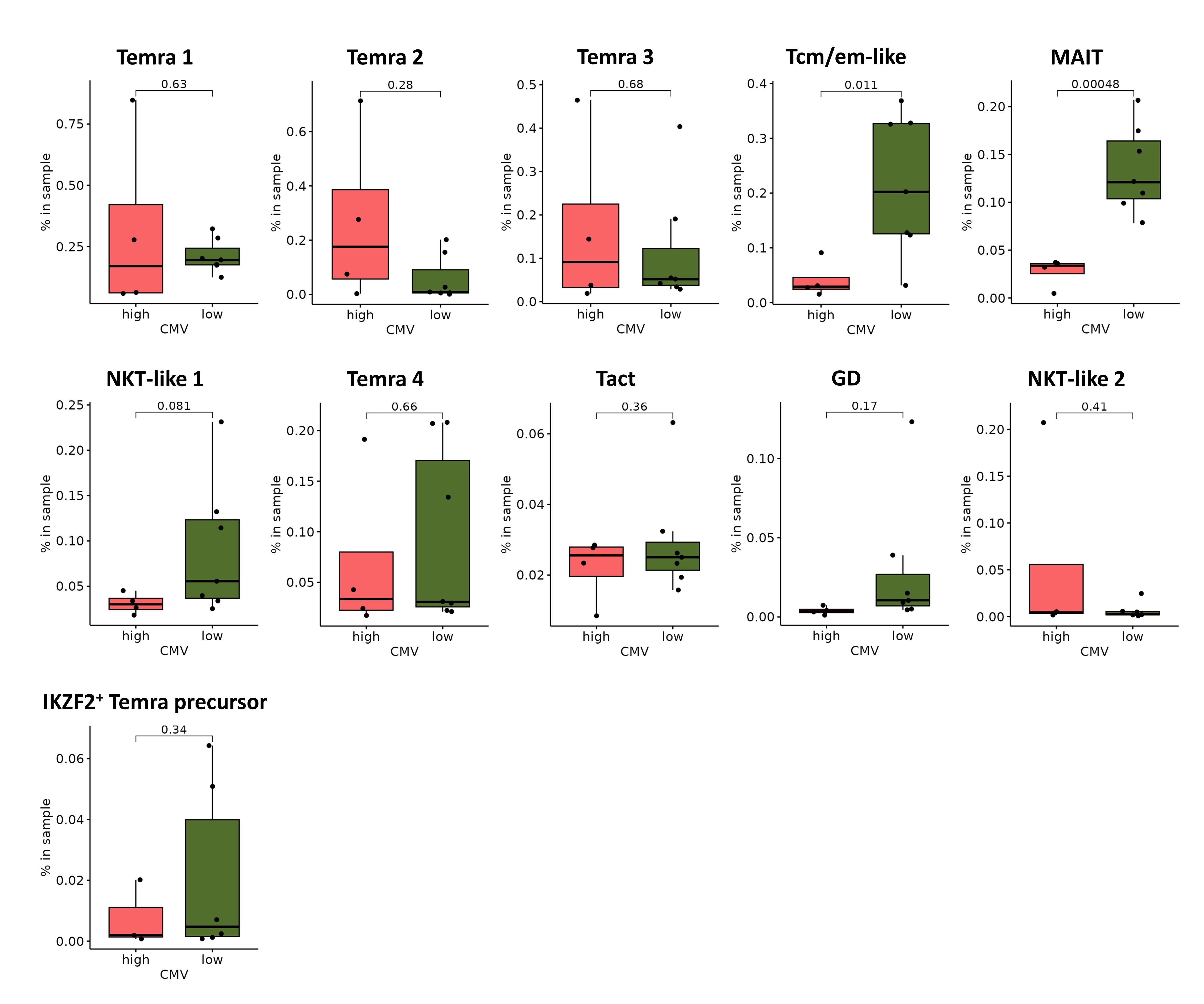

Supplement: Supplementary Figure 12 — Boxplots showing the percentage of cells of each CD8+ T cell cluster between CMVhi and CMVlo. [file Image_12.jpeg]

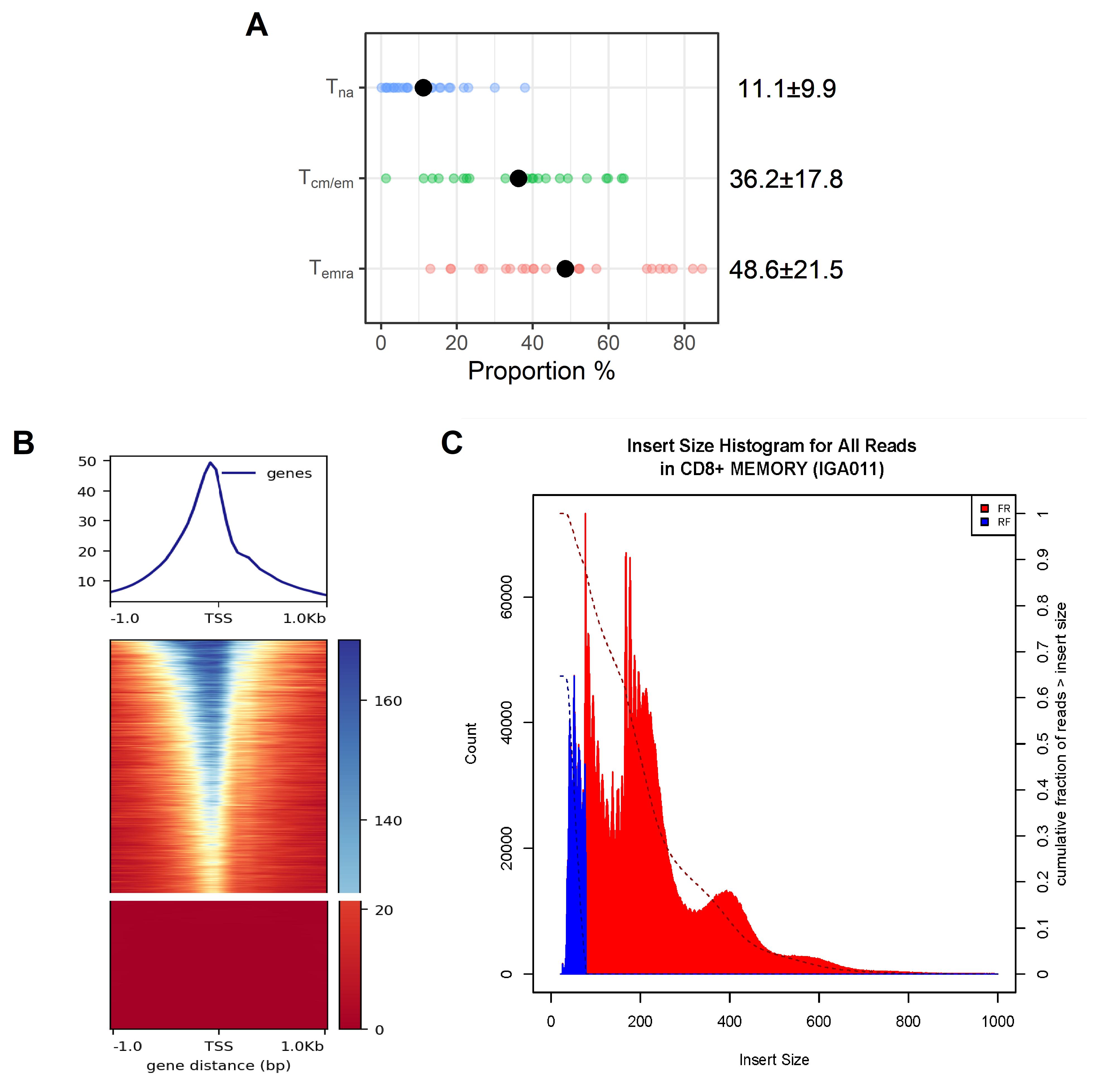

Supplement: Supplementary Figure 13 — Proportions of T cell subsets and quality control of ATAC-seq data. (A) Percentage of sorted CD8+ T cell subsets. Black dot on the graph represents mean. (B) Heatmap summarizing ATAC-seq coverage from one sample. (C) Fragment Size Distribution of one sample. Tna, naïve T cells,; Tcm/em, central and effector memory T cells; Temra, terminally differentiated effector memory T cells. [file Image_13.jpeg]

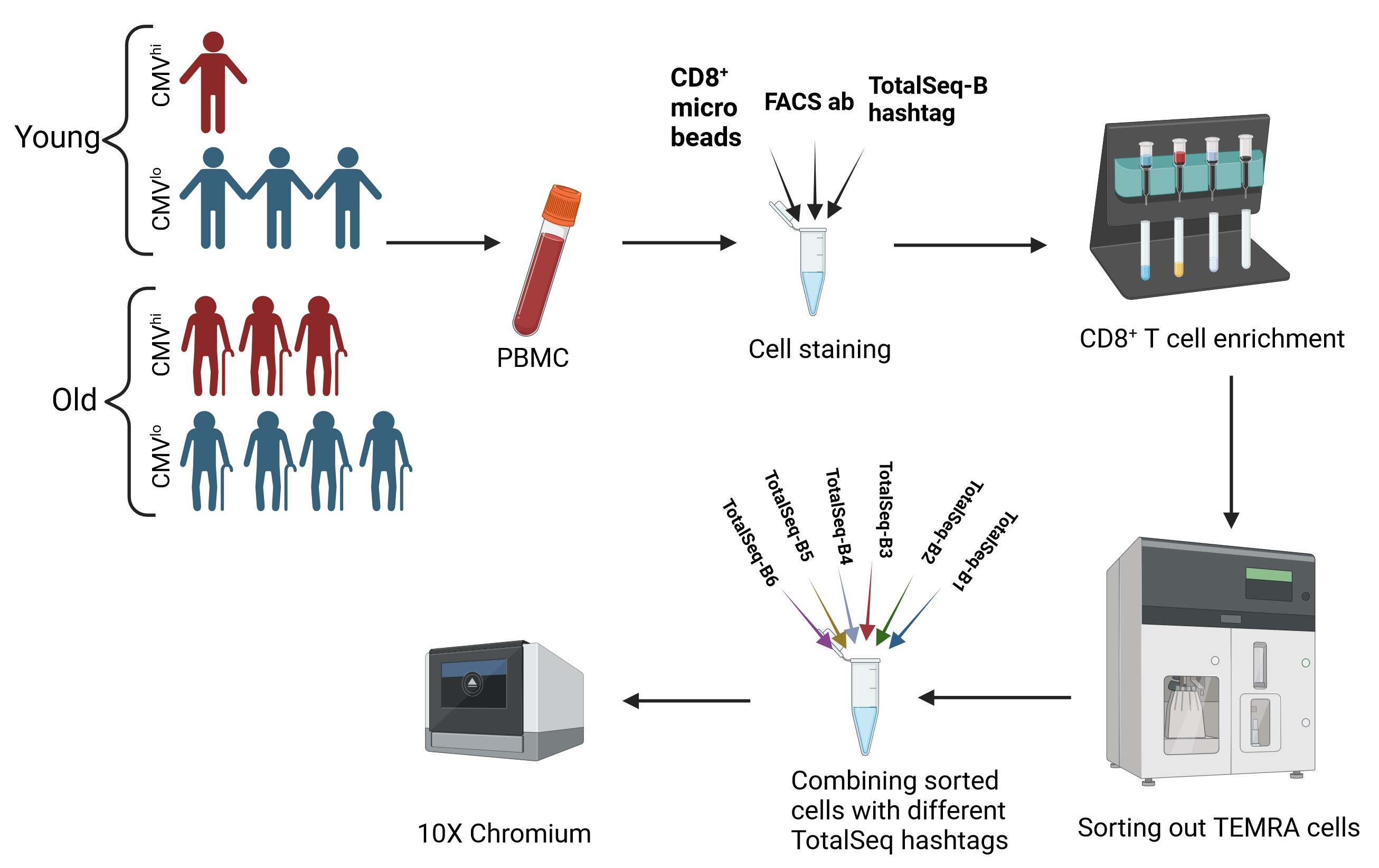

Supplement: Supplementary Figure 14 — Overview of the single-cell study design. For single-cell analyses 4 young and 7 elderly individuals were recruited. After the extraction of PBMCs, cells were stained simultaneously with TotalSeq-B Hashtags, CD8 Microbeads and FACS antibodies. Next, CD8+ cells were extracted using MS columns and MACS separator. Temra cells were sorted using Sony MA900 Cell Sorter. 5000 Temra cells from 5-6 individuals with different barcodes were combined into one reaction and loaded onto the Chromium controller. The image was created using BioRender.com. [file Image_14.jpeg]
